# From tablet to table: How augmented reality influences food desirability

**DOI:** 10.1007/s11747-022-00919-x

**Published:** 2022-12-27

**Authors:** William Fritz, Rhonda Hadi, Andrew Stephen

**Affiliations:** Marketing at Saïd Business School, University of Oxford, Park End Street, Oxford, OX1 1HP UK

**Keywords:** Augmented reality, Mobile technology, Food consumption, Mental simulation

## Abstract

Augmented reality (AR) technology has generated enormous industry investment and buzz, with the food and beverage sector quickly embracing this technology in an effort to enhance the customer experience. However, academic research has only just begun to empirically explore how and why this technology might influence consumer judgements and behaviors in such contexts. Across two field studies involving consequential behavior and two controlled laboratory studies, we find that AR’s unique ability to visually superimpose objects onto a real-time environment increases consumers’ ability to mentally simulate consuming a pictured food, which in turn increases their desire and purchase likelihood of the food item. Further, we find the increased mental simulation produced by AR is itself preceded and driven by an increased sense of personal relevance of the food items.

**Supplementary Information:**

The online version contains supplementary material available at 10.1007/s11747-022-00919-x.

Augmented Reality (AR), technology that superimposes digital content onto real-time physical environments (Tan et al., 2022), has generated enormous amounts of industry investment and buzz, with $200 billion projected to be invested into the development and advancement of AR technologies by 2025 (Liu, 2020). Unlike Virtual Reality (VR), which typically requires standalone headsets, AR simply requires a camera-equipped smartphone or tablet and allows the user to see virtual objects overlaid in the real world (Ko et al., 2013). This accessibility has fueled the widespread popularity of AR applications (including Pokémon Go, Snapchat lenses and Instagram filters; Slater, 2019; Snap Inc., 2020; Tassi, 2018) and has encouraged brands across various industries to explore how AR could potentially be harnessed to influence consumers’ decision-making processes and improve shopping experiences.

Interestingly, one domain which has been particularly quick to experiment with AR technology is the food and beverage industry. Both large corporate fast-food chains (including Domino’s Pizza, Dunkin Donuts, Subway and Panera Bread; QReal, 2019) and small independent dining establishments are experimenting with AR for commercial and consumer-engagement purposes. For example, Domino’s Pizza teamed up with AR developer QReal to create a “shoppable AR” lens for Snapchat, allowing users to see a floating pizza through their camera for direct ordering (Swant, 2018). In a similar vein, Backyard Betty’s restaurant in Boston launched AR versions of many of their menu items, giving customers a QR code to scan and subsequently view dishes at their table prior to ordering (McKinnon, 2018). Many industry leaders believe this is just the beginning for AR food applications, due in part to advancements made by major tech platforms including Snapchat, Facebook, Apple, and Google (Alarcón, 2019). However, some industry voices have expressed concerns that AR’s marketplace potential has been over-hyped, pointing to logistical hurdles and slow rates of consumer adoption (Sullivan, 2021, WIRED, 2021).

Despite the great degree of industry interest and speculation, academic research has only just begun to systematically explore how AR might actually influence consumers’ judgements and behaviors. Much of this research has documented the optimal configurations and settings within AR applications (e.g., considering different levels of customization or interactivity; Carrozzi et al., 2019; Heller et al., 2019a, b; Hilken et al., 2017; Hopp & Gangadharbatla, 2016). While such research has provided valuable insights, our work instead focuses on how AR technology itself—specifically the fundamental ability to superimpose digital stimuli onto consumers’ real-time environment—can influence behavioral responses, with an emphasis on documenting the underlying psychological mechanisms driving any such effects.

Specifically, we empirically examine how AR presentation can influence consumers’ desire and purchase likelihood of depicted foods. We focus on the domain of food for two primary reasons. First, as previously mentioned, the $281 billion (Wunsch, 2021) food and beverage industry has been particularly quick to embrace the potential of this new technology, experimenting with how AR might be used to enhance the decision-making process and dining experience both at restaurants and in the home. Thus, methodically understanding how consumers respond to AR applications in this domain is of substantive importance. However, our focus on food is also theoretically motivated. Specifically, prior research has shown that a critical antecedent for food craving and evaluation is consumers’ ability to engage in mental simulation (e.g., Elder & Krishna, 2012; Hildebrand et al., 2019), a cognitive process particularly influential within highly sensorial product categories (MacInnis & Price, 1987). Consequently, food is an especially ripe domain for investigating the effects of AR-driven mental simulation and represents an opportune realm to begin exploring this technology’s potential influence.^1^

We propose and demonstrate across two field studies involving real choice and purchase data and two laboratory studies that because AR can visually superimpose objects onto a consumer’s real-time environment (via a camera-enabled mobile device; Moro et al., 2017), this visual impression increases a consumer’s ability to mentally simulate consuming the pictured food, which in turn increases the food’s desriability and purchase likelihood. We also show that the increased mental simulation produced by AR is itself preceded and driven by an increased sense of personal relevance for the depicted food items. Importantly, we demonstrate that these effects hold across indulgent, non-indulgent, desirable, and undesirable food categories.

The contribution of the current work is largely substantive in nature (providing a “substantive contribution via deduction;” Lynch et al., 2012). That is, motivated by a real-world phenomenon (the pervasive usage of AR in the marketplace—particularly in the food domain), we rigorously examine how this technology influences consumers and provide evidence that allowing consumers to view products in AR may in many cases be a worthwhile investment for food establishments and brands.

In addition, we make several theoretical contributions by identifying the psychological mechanisms underlying the effects. Specifically, we contribute to the literature on mental simulation by demonstrating and measuring how AR’s ability to visually superimpose products onto a consumer’s real-time environment is uniquely able to generate a sense of personal relevance that elicits mental simulation above and beyond visual stimuli that is not superimposed. In doing so, our work also adds to the growing body of consumer-technology research in marketing that explores how technological features of mobile devices (in this case, a smartphone or tablet-embedded camera) can meaningfully alter the ways consumers behave in today’s marketplace.

## Conceptual framework

To build a conceptual framework for how AR technology might systematically influence consumer responses to depicted foods, we first describe the technology itself and recent explorations into how it might influence consumer behavior, before integrating relevant literature on mental simulation to build our theoretical framework.

### Augmented reality technology

Augmented reality (AR) technology transforms a user’s visual experience of the physical world in real-time, by allowing the user to, “see the real world, with virtual objects superimposed upon or composited with the real world,” (Azuma, 1997). While precise definitions of AR technology vary (Höllerer & Feiner, 2004; Liao, 2016, 2019), most scholars agree that AR is fundamentally characterized by one integral component—real-time superimposition. In current AR applications, real-time superimposition is typically achieved by using a mobile device’s camera to visually recognize one’s immediate environment, on which digitally rendered images are instantaneously overlaid (Athsani & Kalaboukis 2012; Yim et al., 2017; Oh & Bailenson, 2017). Today, consumers are able to engage with AR technology through various devices (including stationary computers and headsets), but the overwhelming majority access AR applications on mobile devices (e.g., smartphones or tablets; Tankovska, 2020). Such applications typically use either the device’s front-facing camera (to project visual content such as make-up or personal accessories onto a user’s face) or the device’s rear-facing camera (to project visual content into the user’s current space). Regardless of the format, the technology is typically used to complement, supplement, or enhance the surrounding physical world with added visual information.

Although sometimes used interchangeably (albeit incorrectly), it should be noted that AR is both theoretically and practically distinct from Virtual Reality (VR). VR is a technology which entirely immerses the user in an artificial, virtually simulated world (Schmitt, 2019; Tan et al., 2022). One way in which we conceptualize the distinction between AR and VR is by understanding the primary function of each technology: superimposition and transportation, respectively. Superimposition, one of the primary functions of AR, is the process of placing, or laying, something on top of directly viewed real-world scenes so that the two coexist and are both still evident (Milgram et al., 1994). This process utilizes the real-world surroundings as a natural backdrop upon which virtual elements are overlaid, providing the viewer with the advantage of visualizing virtual objects “without the vulnerability of being blind to the real world” (Tan et al., 2022), as is the case with VR. On the other hand, transportation, one of the primary functions of VR, removes many real-world sensations (e.g., obstructing the view of the real world via a head-mounted display) and “transports” individuals to another place that may or may not exist in reality (Sadowski & Stanney, 2002; Tan et al., 2022). Put differently, AR leverages technology to visually alter the user’s immediate environment, whereas VR leverages technology to visually remove the user from their immediate environment and transport them to a different, completely synthetic environment, and these different processes will likely result in distinct user outcomes (see Hilken et al., 2022b for an empirical comparison of AR and VR in an experiential retail setting).

Some scholarly work in marketing has begun to explore the psychological implications of AR-enabled visualizations. In Table 1 below, we highlight a selection of papers that have empirically examined the effect of AR on consumer responses, making particular note of the specific manipulations employed and the contexts in which AR was applied. Notably, the bulk of such work largely falls into two categories. The first body of work examines how specific configurations and settings within AR applications influence consumer responses. For example, scholars have examined aspects including the level of customization (e.g., the ability to personalize the visual content; Carrozzi et al., 2019), degree of interactivity (e.g., the ability to manually manipulate or transform the content; Heller et al., 2019a), sensory modality of control (e.g., touch vs. voice, Heller et al., 2019b), exposure time (e.g., how long the user spends on the app; Hopp & Gangadharbatla, 2016) and product composition (e.g., bundled vs. individual, Hilken et al., 2022a).

**Table 1:** Selection of papers empirically examining the effect of AR on consumer responses

Authors	Journal	Camera Direction	Product Category	Device	Core Manipulation	Process Variables	Dependent Variables	Key Findings
*AR Configuration*								
Carrozzi et al. (2019)	*Journal of Interactive Marketing*	Rear-facing	Automotive	Microsoft HoloLens	Customization: low vs. high	- Assimilation - Differentiation	Psychological Ownership	- Customization of AR holograms increases users’ psychological ownership of digital products
Heller et al. (2019a)	*Journal of Retailing*	Rear-facing	Food, Home Decor, Toys	Mobile	Imagery Transformation: low (static) vs. high (dynamic)	- Processing Fluency - Decision Comfort	- Choice - Word of Mouth (WOM)	- High transformation ability in AR improves decision comfort and WOM
Heller et al. (2019b)	*Journal of Retailing*	Rear-facing	Home Decor	Microsoft HoloLens	Sensory Control Modality: Touch vs. voice	- Mental Intangibility - Decision Comfort	Willingness to Pay (WTP)	- Touch control (vs. voice control) increases WTP in AR applications
Hilken et al. (2020)	*Journal of the Academy of Marketing Science*	No camera usage specified (pre-existing images and videos used as stimuli)	Home Decor	Desktop	-POV Sharing Format: static image vs. dynamic video -Communication Style: text-only vs. image-enhanced	Social Empowerment	-Recommendation Comfort - Choice - Desire for Product - Product Usage - WOM	- Static (vs. Dynamic) POV sharing and Image-Enhanced (vs. Text-Only) communication increases recommendation comfort
Hilken et al. (2022a)	*Journal of Interactive Marketing*	Rear-facing	Food Retail	Desktop & Mobile	-Imagery: static 2D picture vs. 3D AR objects -Product Presentation: bundled vs. individual	Self-projection	Purchase Intention	-Viewing bundled products (vs. individual products) in AR increases intended and real purchase
Hilken et al. (2022b)	*Psychology & Marketing*	Rear-facing	Food Retail	Desktop	Technology: (control vs. AR vs. VR)	-Product-focused Imagery -Context-focused Imagery	-Purchase Intention -Brand Attitudes	-AR (vs VR) increases purchase intentions, driven by product-focused imagery -VR (vs. AR) increases brand attitudes, driven by context-focused imagery
Hoffmann et al. (2022)	*Journal of the Academy of Marketing Science*	Rear-facing	CPG	Mobile	-Information Control (controllable vs. uncontrollable) -Information Detail (detailed vs. nondetailed)	-Perceived Comprehensiveness -Perceived Credibility -Perceived Complexity -Perceived User Friendliness	-Brand Image -Purchase Intention -Purchase	-Controllable (vs. uncontrollable) information reduces perceived complexity -Controllable (vs. uncontrollable) information decreases perceived comprehensiveness of the information
Hopp and Gangadharbatla (2016)	*Journal of Current Issues and Research in Advertising*	Front-facing	Automotive	Desktop	Exposure Time: 3 vs. 5 vs. 7 min	Attitude toward AR	Brand Attitude toward	- Novelty was negatively related to Attitude toward AR - High Technological Self-Efficacy transferred negative Attitudes towards AR to the Brand
*Self as backdrop for AR (virtual try-on)*								
Hilken et al. (2017)	*Journal of the Academy of Marketing Science*	Front-facing	Eyewear (virtual try-on)	Desktop	- Physical Control: low vs. high - Environmental embedding: low vs. high	- Spatial Presence - Decision Comfort - Utilitarian/ Hedonic Value	- Purchase - Word of Mouth	- AR-based service augmentation enhances customer value perceptions
Poushneh and Vasquez-Parraga (2017)	*Journal of Retailing and Customer Services*	Front-facing	Eyewear (virtual try-on)	Desktop	Level of Interactivity: high vs. middle vs. low	User Experience (UX)	- User Satisfaction - Willingness to Buy	- High and middle interactivity positively influences UX
Smink et al. (2019)	*Electronic Commerce Research and Applications*	Front-facing	Cosmetics (virtual try-on)	Desktop	Presentation: dynamic-self vs. static-self vs. static-other	- Perceived Informativeness - Perceived Enjoyment - Perceived Intrusiveness	- Brand Attitude - Purchase Intention - Willingness to Share Personal Data	- Dynamic-self enhances Perceived Informativeness and Enjoyment - Informativeness increases Purchase Intentions and Willingness to Share Personal Data - Enjoyment increases Brand Attitude
Tan et al. (2022)	*Journal of Marketing*	Front-facing	Cosmetics (virtual try-on)	Mobile	Pre- vs. post- AR Availability (analysis of secondary dataset)	Product Characteristics: - Brand Popularity - Product Appeal - Product Rating - Product Price Customer Characteristics: - New to Channel - New to Category	Sales	- AR (vs. no-AR) is associated with higher sales for brands that are less popular, products with narrower appeal, and expensive products - Effect of AR is stronger for customers new to the channel and category
Yim et al. (2017)	*Journal of Interactive Marketing*	Front-facing	Eyewear, Watches (virtual try-on)	Desktop	Media: website viewing vs. AR try-on	- Immersion - Media Usefulness - Enjoyment	- Attitude toward AR - Purchase Intention	- AR’s Interactivity and Vividness increases users’ sense of immersion (compared to a desktop website), which increases enjoyment, usefulness, and attitude towards AR
*Other AR Research in Marketing*								
Jessen et al. (2020)	*Journal of Business Research*	Rear-facing	Interior Design	Mobile	Design Location: Desktop room-rendering vs. AR in physical room)	- Customer Engagement - Customer Creativity	Anticipated Satisfaction	- Mobile AR Design (vs. Desktop Design) increases anticipated satisfaction with purchase decisions
Yaoyuneyong et al. (2016)	*Journal of Interactive Advertising*	Rear-facing	Retail	Mobile	Supplemental Information to Print Ad: none vs. QR code vs. AR text	N/A	Attitudes toward Advertisement	- AR Ad resulted in highest perception of informativeness, novelty and effectiveness - QR Ad resulted in higher irritation - Traditional Print-Ad resulted in higher time-effort

The second category of work has almost exclusively focused on “virtual try-on” experiences, which typically involve a front-facing camera that superimposes clothing, accessories, or makeup onto the users themselves (Hilken et al., 2017; Poushneh & Vasquez-Parraga, 2017; Smink et al., 2019; Tan et al., 2022; Yim et al., 2017). Collectively, such work has found that AR can improve consumer responses, including brand attitudes and purchase intentions. While this body of work does involve a form of visual superimposition, it represents a practically and theoretically unique area of investigation, given that such applications superimpose digital content onto the users *themselves* (as opposed to superimposing content into the user’s environment/space). Thus, AR presentation in such contexts necessarily introduces the confound of simultaneously providing a visual portrayal of the user themself (a factor that previous research has found to have significant effects on consumer attention and attitudes—e.g., Chang and Hung 2018; Cho and Schwarz 2010; Hung and Wyer 2011).

Our research complements and builds upon the existing work in at least two ways. First, as opposed to exploring specific configurations or settings within AR applications (e.g., level of customization or degree of interactivity), we hold such aspects constant, instead empirically isolating and focusing on AR’s fundamental ability to superimpose digital objects onto a consumer’s real-time environment. In other words, we explore whether and how such superimposition of visual content can, in and of itself, influence consumers’ evaluations and purchase likelihood.^2^ Second, we complement extant work by exploring and identifying the underlying psychological mechanisms that might explain such effects. To provide support for our theorizing, we next turn to research on mental simulation.

### Visually induced mental simulation

We focus our empirical investigation within the food domain for both substantive and theoretical reasons, as previously mentioned. Notably, academic research has found that judgments and decision-making with respect to food are often influenced by a consumer’s propensity to engage in *mental simulation,* particularly by a consumer’s tendency to imagine consuming a designated food (Elder & Krishna, 2012; Hildebrand et al., 2019; Kappes & Morewedge 2016). Neuroscience research has shown that mental simulation can engage parts of the brain associated with tasting, smelling, and hearing stimuli (Krishna, 2012; Schifferstein, 2009), and several consumer researchers have accordingly shown that when consumers mentally simulate (i.e., imagine) consuming a food item, it increases their immediate desire for it (Elder & Krishna, 2012; Hildebrand et al., 2019). While mental simulation has also been shown to improve responses to non-food product categories, research suggests it is most beneficial for highly sensorial and hedonic (as opposed to utilitarian) product attributes (MacInnis & Price, 1987).

Mental simulation can be induced from all sensory modalities, but visual images tend to be perceived the most vividly and therefore are the method most commonly used to induce mental simulation (Schifferstein, 2009). This may have evolutionary roots, as our sense of sight at least partially developed in order to increase our species’ chances of survival by identifying the most nutrient and energy-rich sources of food (Spence et al., 2016). Yet importantly, not all visual imagery is equally likely to induce mental simulation. For example, researchers have found differences across high quality versus low quality pictures (Petrova & Cialdini, 2005; Rossiter & Percy, 1980) and dynamic versus static images (Lutz & Lutz, 1977, 1978; Roggeveen et al., 2015; Schlosser, 2003). Most relevant to the current research is work finding that *contextual* visual cues can also play an influential role in inducing mental simulation. For example, Hildebrand et al. (2019) demonstrate that displaying foods with an occasion-setting background (e.g., depicting a pizza over a depiction of a pizzeria vs. a solid or incongruent background) can increase mental simulation tendencies, especially for holistic thinkers. Similarly, Elder and Krishna (2012) show that subtly portraying food in a manner more fluent with consumption (e.g., visually placing a fork on the same side as the viewer’s dominant hand) can similarly induce greater mental simulation of consumption.

Given that AR has the ability through superimposition to create vivid illusions of a product’s presence in a user’s immediate real-world environment, we expect AR technology has a high potential to induce mental simulation in consumers. In fact, at the conclusion of their meta-analysis on mental simulation, Ceylan et al. (2022) speculate that augmented imagery might facilitate mental simulation, and explicitly invite future research to explore this phenomenon.

Notably, while much research has treated mental simulation as a unidimensional construct, some work has added nuance to the mental simulation literature by distinguishing between two distinct types of mental simulation, each of which have been shown to influence consumers’ judgments and behavior: process-focused simulations and outcome-focused simulations, respectively (Castaño et al., 2008; Escalas & Luce, 2004; Ringler et al., 2021; Taylor et al., 1998; Zhao et al., 2011). Process-focused simulations, or “how-thinking,” makes salient the process of engaging in an activity through the use of sensory cues to influence product evaluations and behavioral outcomes, including willingness to pay, purchase intention, goal completion, and consumption (Castaño et al., 2008; Escalas & Luce, 2003; Ringler et al., 2021; Taylor et al., 1998). Conversely, outcome-focused simulations, or “why-thinking,” makes salient the outcome from engaging in an activity, without consideration for how that outcome was achieved (Castaño et al., 2008; Escalas & Luce, 2003, 2004). We expect that the mental simulation facilitated through AR presentation will most closely resemble process-focused simulation rather than outcome-focused simulation, as the superimposition facilitated by AR provides users with the opportunity to imagine “engaging” with, or consuming, the food item that has been projected in their immediate space, as opposed to simply imagining a post-consumption outcome (e.g., feeling full, satiated, or any other type of consumption consequence).

### The current research: How AR influences food desirability

As previously discussed, much of the recent marketing research on augmented reality has focused on *how* consumers interact with AR applications (Tan et al., 2022). These explorations have included manipulations of customizability, interactivity, modality of control, and exposure time (Carrozzi et al., 2019; Heller et al., 2019a, 2019b; Hilken et al., 2020; Hopp & Gangadharbatla, 2016). While such research has provided valuable insights into the optimal configuration of AR, our work instead focuses more fundamentally on how one critical aspect of AR technology itself—the superimposition of digital stimuli onto consumers’ real-time environment—can influence behavioral responses. Motivated by both managerial prevalence as well as prior research establishing the link between visual contextual cues and increased mental simulation in the context of food consumption (e.g., Elder & Krishna, 2012; Hildebrand et al., 2019), we focus our investigation on the effects of AR-induced mental simulation in this consequential and highly-sensory domain. This focus addresses the recent call by Tan et al. (2022) to explore how AR can most effectively be leveraged by the service and hospitality sectors and how this technology may influence consumers’ judgments and decisions.

Given that AR has a unique ability to visually superimpose digital objects on top of one’s real-time visual environment, together with the knowledge that visual contextual cues often serve as critical antecedents of mental simulation (e.g., Elder & Krishna, 2012; Hildebrand et al., 2019), which itself has been shown to increase food desirability and purchase intentions (particularly within highly sensorial product categories; MacInnis & Price, 1987), we formally hypothesize:

- **H1** Using AR to visually superimpose depicted food items onto a consumer’s real-time environment will (a) increase desirability; and (b) improve purchase likelihood, relative to depicting the food item in a non-superimposed format.
- **H2** The positive effects of AR presentation on food desirability and purchase likelihood will be driven by increased mental simulation.

While the effects of mental simulation on downstream variables have been well-documented (albeit not in the AR context) in extant literature (Ceylan et al., 2022; Elder & Krishna, 2012; Hildebrand et al., 2019; MacInnis & Price, 1987), little research thus far has explored the link between AR technology and mental simulation. Therefore, it is compelling to consider what specifically about AR might increase consumers’ mental simulation in the first place. One possible explanation comes from AR’s unique ability to visually superimpose a virtual object onto a consumer’s peripersonal space (the immediate space around one’s body which can be touched or manipulated, Holmes & Spence, 2004). Objects which appear in one’s peripersonal space are likely to be perceived as personally relevant, since people tend to surround themselves with objects they enjoy and that are of personal relevance, rather than objects which are not personally relevant. Therefore, it is plausible that by superimposing an object into one’s peripersonal space via AR, that object should be perceived as more personally relevant to the individual, compared to the same object that is not superimposed into one’s peripersonal space. Further, perceived personal relevance of an object or advertisement has been previously shown to increase both message processing (Ajzen et al., 1996) and mental simulation (Buckner et al., 2008; Gutsell & Inzlicht, 2010; Ülkümen & Thomas, 2013). Notably, Ülkümen and Thomas (2013) specifically demonstrate that messages framed with high personal relevance (versus low personal relevance) led participants to spontaneously simulate the action of the message (i.e., process-oriented mental simulation). Stringing these findings together, we argue that the superimposition of a virtual object into a users’ peripersonal space should increase the perceived personal relevance of that object, which in turn will increase the ease of mentally simulating engaging with the superimposed object. Formally, we hypothesize:

- **H3** The positive effect of AR superimposition on mental simulation will be driven by increased perceived personal relevance of the virtual object.

**Fig. 1:**
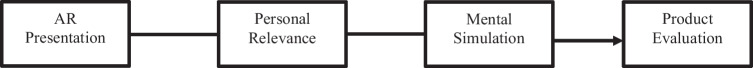
Proposed conceptual model

Our theorizing is collectively illustrated in Fig. 1 below. Notably, while AR can be presented in a variety of forms, we focus the current investigation on its manifestation through mobile devices, given their ubiquity and dominance as the primary form factor for consumer-facing AR applications (Tankovska, 2020). Behavioral research in marketing has explored how mobile devices can systematically influence consumers’ attitudes and behaviors (e.g., Bart et al., 2014; Grewal & Stephen, 2019; Melumad et al., 2019, Melumad & Meyer, 2020; Song and Sela, 2022), and some work has focused on how specific features of mobile devices play a role. For example, scholars have demonstrated that the touchscreen feature on these devices can influence both psychological ownership and consumer choices (Brasel & Gips, 2014; Shen et al., 2016). Other work by Hadi and Valenzuela (2020) has shown device-delivered haptic feedback can improve consumer responses to communications. Diehl et al. (2016) explored how the camera function on mobile phones allows consumers to increase their enjoyment experiences through increased photo-taking. Our research expands on this prior work by exploring how another unique capability of mobile devices, AR presentation using the integrated camera, can alter and drive consumer responses.

### Overview of studies

We test our conceptual model (Fig. 1) and hypotheses across four experimental studies,^3^ which collectively provide empirical support for our proposed theorizing across both large (e.g., tablets; Studies 1–3) and small (e.g., smartphones; Study 4) mobile devices, in the context of indulgent (i.e., dessert in Studies 1 and 2), non-indulgent (Study 3), and both desirable and undesirable (Study 4) food categories. Notably, the first two studies were conducted in field settings to lend external validity to our investigation, while the lab-based nature of the subsequent two studies allowed us to achieve rigor in our measurement of psychological processes. Study 1, a field experiment run at a restaurant, demonstrates that presenting indulgent foods in AR (versus a non-superimposed format) can increase both desirability and consequential downstream variables (i.e., real purchase), supporting H1. In Study 2, a field experiment run at a café, we replicate the positive effect of AR presentation and provide preliminary support for mental simulation as the underlying process, hence supporting H2. In Study 3, conducted in a behavioral lab, we replicate the effect of AR presentation on desirability and purchase likelihood of a non-indulgent food item, use multi-item measures to more robustly support the mediational role of mental simulation, and additionally find support for personal relevance as an anteceding mediator (supporting H3, and fully testing the model illustrated in Fig. 1). In the final study (conducted in a behavioral lab), we find converging support for our overarching model (Fig. 1) using a smaller and more accessible mobile device (i.e., smartphone), extend the generalizability of our findings (by demonstrating that the results hold for both desirable and undesirable food items), and more precisely identify the type of mental simulation at play (i.e., process-oriented mental simulation).

## Study 1: A field study of the effect of AR presentation on food desirability and purchase

The main purpose of Study 1 was to examine the effect of AR presentation on food desirability and purchase behavior (testing H1). This study was a field experiment, run with the cooperation of a restaurant in a large international city. Specifically, we created an AR version of the restaurant’s existing dessert menu, which allowed us to assess whether diners who viewed the menu in an AR format were more or less likely to purchase a dessert as compared to diners who viewed the menu in a non-superimposed (but still digital) format.

### Design and procedure

One hundred and one diners^4^ (41% female, 59% male, 0% nonbinary/other; M_Age_ = 37.95, SD = 13.04) participated in exchange for a £5 discount on their restaurant bill. The experiment used a two-level (presentation format: control versus AR) between-subjects design. We ran this experiment in collaboration with a brasserie-style restaurant in a large international city (see Appendix A for photographs of the restaurant). To manipulate presentation format, we created two versions of the restaurant’s existing paper-based dessert menu (six items, see Appendix B for a list of all options on the dessert menu). For the control condition, we took high-resolution photographs of each dessert, and presented these as a digital menu on a tablet (an Apple iPad) given to diners. For the AR condition, we used a professional 3D scanning photogrammetry kit to take over 400 high-resolution photographs of each dessert from various angles. These images were then given to a professional AR developer (QReal) for conversion into 3D renderings of each dessert that were viewable as AR objects using a mobile app installed on the tablet.

Importantly, while both menu formats were electronic and viewed in a mobile app on a tablet, the control menu displayed two-dimensional desserts on static blank backgrounds, whereas the AR menu displayed three-dimensional desserts superimposed in a diner’s environment (i.e., on the restaurant table in front of them). The two presentation formats are illustrated in Appendix C (while the AR condition included a manipulation of both dimensionality and superimposition in this study, our next two studies attempt to separate these factors).

The experiment was run on two consecutive weekday evenings during the dinner shift (6 pm – 10:30 pm). After restaurant diners completed the main course of their meal, an experimenter blind to the hypothesis and posing as a waiter approached their table with a tablet and informed them that the restaurant had created a “digital dessert menu” and was offering diners a discount off their bill for simply looking at the menu and providing feedback (without any obligation to order anything if they did not wish to).

Each table was randomly assigned to view the dessert menu in either the control or AR format on an alternating basis (we adopted this procedure as opposed to random assignment at the individual level to prevent diners at the same table from becoming aware of the manipulation). While viewing the menu, participants completed a paper survey in which they assessed the desirability of each dessert on a 7-point scale (1 = “very undesirable” to 7 = “very desirable”). After placing their dessert orders (if any) with the experimenter, participants were asked their age, gender, and familiarity with augmented reality technology (on a 7-point scale, 1 = “very unfamiliar” to 7 = “very familiar; for complete list of all measures used in Study 1, see Web Appendix B). Afterwards, all diners were served any desserts they ordered. At the end of the meal, the experimenter collected the corresponding receipts for each table, which indicated all food items and beverages ordered, how much money was spent, and the number of people at each table.

### Results and discussion

#### Dessert Purchase

A binary logistic regression found that participants were significantly more likely to order a dessert if they viewed options in the AR menu (41.2%) versus the control menu (18.0%; Wald χ^2^ (1) = 6.21, *p* = 0.01). Controlling for the number of people at each table and the average money spent per person strengthened this effect (Wald χ^2^ (1) = 9.50, *p* < 0.01).^5^ Presentation format did not significantly influence choice share of any particular dessert relative to others (all *p*’s > 0.15). While not germane to our investigation, it is worth noting that the number of people at the table did exert a significant and negative main effect on participants’ likelihood to order dessert (*B* = -0.622; *p* < 0.01),^6^ but importantly, this variable did not significantly interact with AR presentation (*p* > 0.98).

As a robustness check, we also calculated the average amount of money each diner spent on dessert (including participants who did not spend any money on dessert). ANOVA results demonstrated that those in the AR menu condition spent significantly more on dessert than those in the control condition (M_Control_ = £1.38 versus M_AR_ = £2.93; *F*(1, 99) = 7.58, *p* < 0.01, η_p_^2^ = 0.07). Receipts from the week following our data collection suggest that the typical likelihood of a restaurant diner ordering dessert at this restaurant (ordered from a print-based menu) on a weekday evening is 7%. The increased likelihood of dessert purchase in our study (in both presentation format conditions) is likely due in part to the fact that diners were given a discount off their bill for looking at the menu. Importantly however, this cannot explain the difference we find across the control and AR conditions.

While the menus in both conditions were digitally presented on tablets, one could still reasonably argue that the AR menu format was more novel than the control menu format. Accordingly, to examine whether novelty might explain the positive effect of AR presentation on purchase likelihood, we adopted a procedure from previous literature (Heller et al., 2019a; Venkatesh et al., 2012) by examining whether downstream responses to presentation formats differed according to diners’ familiarity with AR technology. AR familiarity did not differ across the two presentation format conditions (M_Control_ = 4.48 versus M_AR_ = 4.41; *F*(1, 99) = 0.03, *p* = 0.87). Importantly, neither the main effect of AR familiarity nor the interaction between presentation format and AR familiarity were significant predictors of purchase likelihood (both *p*’s > 0.26). In other words, diners with both high and low levels of familiarity with AR technology responded the same way to the presentation format, suggesting that it is unlikely that the AR effect observed here can be explained by the novelty of the technology.^7^

#### Desirability

Given that diners rated the desirability of all desserts on the menu, we ran a repeated-measures ANOVA with dessert type as a within-subjects variable and presentation format as a between-subjects factor. Results demonstrated a significant effect of presentation format on dessert desirability (*F*(1, 99) = 26.34, *p* < 0.001, η_p_^2^ = 0.21) in the predicted direction; i.e., the average desirability of the desserts was higher in the AR menu condition than in the control menu condition. Additionally, there was no interaction between presentation format and the within-subject dessert type factor (*F*(1, 99) = 0.06, *p* = 0.81). As was the case with the purchase likelihood dependent variable, controlling for the number of people at each table and the average money spent per person strengthened the AR effect (*F*(1, 97) = 30.17, *p* < 0.001, η_p_^2^ = 0.24).^8^ Importantly, as was the case with purchase likelihood, neither the main effect of AR familiarity nor the interaction between presentation format and AR familiarity were significant predictors of desirability (both *p*’s > 0.25).

#### Ancillary analysis

We also examined whether participants’ age or gender might act as significant covariates in the analyses above. Neither variable was significant in predicting the influence on dessert purchase (both *p*’s > 0.70) or dessert desirability (both *p*’s > 0.30), and our results continue to hold while controlling for these variables (*p* < 0.001 for the effect of presentation format on both dessert purchase and desirability).

In sum, our first study provided initial evidence under naturalistic conditions that presenting foods in AR (versus a non-superimposed format) can increase both desirability and purchase likelihood, supporting H1. Further, we rule out the potential effect of novelty, and demonstrate the effect holds regardless of a viewer’s age or gender. Importantly, the control condition in this study was a non-superimposed two-dimensional image, as this represents an externally-valid depiction commonly used in restaurants. However, to better isolate the role of superimposition specifically, we employed a more conservative control condition in our remaining studies (holding dimensionality constant).

## Study 2: Replication with more conservative control condition and preliminary support for the role of mental simulation

Study 2 served several purposes. First, we sought to replicate the AR effect on desirability, this time using a more conservative control condition allowing us to isolate the effect of superimposition from visual dimensionality, and to accordingly eliminate any confounds stemming from additional information acquisition in the AR condition. Specifically, we held dimensionality constant (i.e., the 3D renderings of the food were identical in both conditions), and we solely manipulated superimposition by varying whether or not the item appeared in the user’s real-time environment. Second, we wanted to begin exploring the underlying process (namely our proposed mediator, mental simulation), to provide a preliminary test of H2. Finally, we took the opportunity to measure participants’ post-consumption enjoyment of food items, allowing us to examine how AR presentation prior to consumption might ultimately influence this important post-consumption response. To maintain a high degree of external validity, we worked with a university catering team to run this field experiment in the café of a business school that was frequented by students and staff.

### Design and procedure

One hundred and thirty participants (composed of business school students and staff) participated in this study in exchange for a free dessert. We used a two-level (presentation format: control versus AR) between-subjects design. For this study, we worked closely with the university catering team that wanted to showcase three new dessert items developed by their chef for the café (see Appendix D for pictures of these desserts). We again used a professional 3D scanning photography kit to take over 400 high resolution photographs of each dessert from various angles and worked with the same AR developer to convert these photographs into 3D renderings. To manipulate presentation format in this study, we again created two versions of a digital dessert menu. This time, both conditions featured food items which were visually identical in every possible way (e.g., in scale, resolution, dimensionality, etc.). In the control condition, the menu featured the 3D renderings of each dessert over a static blank background. In the AR presentation, as in Study 1, the same 3D renderings for the control condition were superimposed onto the viewer’s real-time environment using the tablet’s camera. Accordingly, the only difference across conditions was the background behind the dessert item: the dessert was featured on a static background for the control condition, or the dessert was visually superimposed in real-time in the AR condition. In both conditions, participants were equally able to interact with the stimuli by using their fingers on the touchscreen of the tablet to rotate, reposition, and resize the featured foods as they desired. The two presentation formats are illustrated in Appendix E.

The experiment was run in the café on a weekday afternoon during lunch hours. An experimenter blind to the hypothesis approached students and staff who were seated and appeared to have just finished having lunch and informed them that the catering team was testing new desserts. Participants were offered a free dessert in exchange for providing feedback on a brief paper survey about the new menu items. Participants were then randomly shown either the control or AR digital dessert menus, done on an alternating basis. After viewing the menu, participants were asked to choose the item they would like to receive. They then indicated the desirability of the dessert (“I am craving the dessert I chose”) and responded to a one-item measure of mental simulation (“When viewing the dessert, I could imagine myself eating it,” taken from Elder and Krishna, 2012; we use more comprehensive scales in Study 3), both measured on a 7-point scales (1 = “strongly disagree” to 7 = “strongly agree”). After completing these items, participants were served the dessert they had selected. After consumption, they indicated how much they enjoyed the dessert (“I enjoyed the dessert, measured on a 7-point Likert scale 1 = “strongly disagree” to 7 = “strongly agree”; see Web Appendix C for complete list of all measures).

### Results and discussion

#### Desirability

After 109 participants had completed the study, the kitchen ran out of one of the dessert options (chocolate brownie). Accordingly, the final batch of participants (N = 21) were only given two dessert options to choose from. Results did not demonstrate any difference between the two batches of participants on any of our dependent variables (all *p*’s > 0.25). Accordingly, we did not exclude any participants and simply include participant batch as a covariate in our remaining analysis. ANCOVA results demonstrated a significant effect of presentation format on dessert desirability (M_Control_ = 4.90 versus M_AR_ = 5.46; *F*(1, 127) = 8.40, *p* < 0.01, η_p_^2^ = 0.06) in the same direction as Study 1 (thus adding support for H1): desirability was higher for the chosen dessert when it was viewed in the AR (versus control) menu format. The effect of presentation format did not differ according to which dessert was chosen (*p* = 0.26).

#### Mental simulation

An identical ANCOVA with the mental simulation item as the dependent variable demonstrated a significant effect of presentation format, in that participants were more likely to imagine eating the chosen dessert when they had viewed it in the AR (versus control) format (M_Control_ = 5.49 versus M_AR_ = 5.90; *F*(1, 127) = 5.19, *p* = 0.02, η_p_^2^ = 0.04). To assess whether mental simulation could explain the increased desirability induced by the AR (versus control) presentation, we ran a mediation analysis (model 4 of the PROCESS macro, Hayes, 2018) with 10,000 resamples. Results demonstrated a significant indirect effect (indirect effect = 0.1159, 95% CI: 0.0082 to 0.2816), confirming our predictions and supporting H2.

#### Post-consumption enjoyment

ANCOVA results also demonstrated a significant effect of presentation format on participants’ enjoyment of the dessert upon consuming it (M_Control_ = 5.59 versus M_AR_ = 6.03; *F*(1, 127) = 4.02, *p* = 0.05, η_p_^2^ = 0.03), in that participants who had viewed the dessert in the AR menu pre-consumption reported greater post-consumption enjoyment than those in the control menu condition. While we did not have formal a priori predictions about the effect of AR presentation on post-consumption enjoyment, these results are consistent with previous work showing that savoring an upcoming experience increases enjoyment of that experience both in real-time as it is happening (i.e., during consumption), and when it is remembered later on (Chun et al., 2017).^9^

In sum, our second study provided further evidence under naturalistic conditions that presenting foods in AR (versus a control format) can increase a food’s desirability. Importantly, this study allowed us to isolate superimposition (versus dimensionality) as the fundamental AR characteristic behind this effect. In addition, we provide preliminary evidence that mental simulation is the underlying process explaining the positive effect of AR on desirability, supporting H2. Finally, we demonstrate that AR presentation does not only influence pre-consumption variables (i.e., desirability and purchase likelihood), but can also exert an influence on post-consumption variables (specifically, consumption enjoyment).

Notably, our finding that increased mental simulation improves desirability (i.e., the second path of our proposed theorizing), has already been well-established in extant literature. However, this research is the first to empirically demonstrate that the real-time superimposition afforded by AR can increase mental simulation (the first path in our model). Accordingly, to better understand this relationship between AR presentation and mental simulation, we wished to examine what preceding mediators might explain the link between AR presentation and mental simulation to begin with.

Thus, in our next study, in addition to more definitively and robustly testing the effect of AR on desirability and purchase likelihood as well as the proposed mediating role of mental simulation, we test a number of additional processes that could possibly explain the link between AR and mental simulation or that could serve as alternative processes explanations more generally.

## Study 3: Establishing the role of personal relevance

Results from the previous field experiments established the effect of AR presentation on desirability and purchase likelihood and provided initial evidence of the key role mental simulation plays in this process. Study 3 served several additional purposes. First, while Studies 1 and 2 focused on indulgent desserts, this study allowed us to extend our examination to a non-indulgent food category (lamb shawarma; as verified in the pretest reported in Web Appendix D). In addition, while the field settings of Studies 1 and 2 placed logistical constraints on the number of items we could include in the surveys, the laboratory setting of Study 3 provided the opportunity to collect multi-item measures of our dependent variable and of our proposed mediator (mental simulation; allowing us to more definitely test H2), among other measures.

Further, as mentioned earlier, while the effects of mental simulation on downstream variables have been well-documented (albeit not in the AR context) in extant literature (Ceylan et al., 2022; Elder & Krishna, 2012; Hildebrand et al., 2019; MacInnis & Price, 1987), little is known about how and why AR induces mental simulation to begin with. This study allowed us to explore whether the positive effect of AR presentation on mental simulation and subsequent desirability might be driven by an increase in perceived personal relevance (hence testing H3). Accordingly, this study allowed us to test the complete model proposed in Fig. 1.

Finally, it is possible that alternative, or multiple, processes could explain the link between AR presentation and mental simulation. Therefore, we leant on both extant literature and intuitive logic to compile an extensive list of potential alternative processes to assess. For example, previous research has suggested that consumers may perceive ownership of digitally displayed products (Atasoy & Morewedge, 2018; Brasel & Gips, 2014), and this seemed particularly worth exploring given AR’s ability to superimpose products onto one’s peripersonal space. Work by Elder et al. (2017) shows how imagined sensory experiences vary in psychological distance, which can positively influence product evaluations. Accordingly we examined whether AR might exert an effect on psychological distance (either spatial or temporal distance). Finally, we explore a number of potential processes called for by Wedel et al. (2020) in their recent conceptual framework on AR and VR for consumer marketing, including: presence, attention, fluency, realism, and mood.

### Design and procedure

One hundred and eight volunteers from a university setting (46% female, 54% male, 0% nonbinary/other; M_age_ = 29.73, SD = 6.61) participated in this experiment in exchange for monetary compensation. The study employed a 2 cell (presentation format: control versus AR) between-subjects design. Presentation format was manipulated as in Study 2 (keeping scale, resolution, dimensionality, and interactivity constant): the control condition featured the 3D food item over a static blank background, while the AR condition superimposed the 3D food item onto the viewer’s real-time environment), except this time the target stimuli was a lamb shawarma (see Appendix F for videos and photographs of the stimuli).

This study was conducted during the COVID-19 pandemic in a university that was allowing students to attend classes in person with appropriate protection measures and social distancing in place. To comply with local COVID-19 government regulations, we modified a lecture theater to serve as a laboratory (see Web Appendix E for complete list of precautions taken to ensure participant safety and regulatory compliance). Once participants arrived at the lab, they scanned a QR code with their mobile phone to access a mobile survey that included the consent form and survey questions. Participants were told that they would view and evaluate lamb shawarma on a tablet, but before viewing the item, they were asked to indicate their prior experience with the food (“I have eaten lamb shawarma before*,”* with the options, “yes,” “no,” or “unsure”). Then, all participants were given a tablet to view the lamb shawarma in either the control or AR presentation format, according to their randomly assigned condition. Importantly, due to social distancing requirements, participants were sat far enough apart as to not be able to interact or engage with one another.

Participants responded to survey questions on their mobile phones while continuing to view the lamb shawarma on the tablet (should they wish to). To take advantage of the laboratory setting, participants were asked to respond to a battery of measures (see Web Appendix F for a full list of items). Our dependent variables of interest in this study were desirability (3-item scale including those from Studies 1 and 2; *α* = 0.89) and purchase likelihood (“After viewing the lamb shawarma, how likely would you be to order it if it was offered on a menu?”). Our proposed mediators were personal relevance (2-item index; e.g., “This is similar to other foods I eat”; *r* = 0.56) and mental simulation (6-item scale adapted from Hildebrand et al., 2019; e.g., “I could imagine myself eating the lamb shawarma displayed”; *α* = 0.89). In addition, we measured potential alternative process explanations including: presence (3 items adapted from Slater et al., 1994; e.g., “The lamb shawarma felt like it was on the table in front of me”; *α* = 0.68), attention to background (“I paid more attention to the lamb shawarma than I did the background behind it,”), perceptual fluency (2 items adapted from Labroo et al., 2008, e.g., “It was easy for me to evaluate this food item”; *r* = 0.85), psychological ownership (2 items adapted from Atasoy & Morewedge, 2018; e.g., “I felt like the lamb shawarma was already mine”; *r* = 0.65), spatial and temporal distance (items adapted from Elder et al., 2017; spatial: “How close do you think the restaurant offering the lamb shawarma is located?”or temporal: “How quickly do you think the restaurant could deliver this lamb shawarma to you?”), enjoyment of the experience (2 items, e.g., “It was fun to view this item”; *r* = 0.77), and realism (4 items, e.g., “This lamb shawarma looks real”; *α* = 0.86). Finally, participants indicated their mood (2 items, e.g., “I am in a good mood right now”; *r* = 0.67), familiarity with AR Technology (as measured in Study 1), gender and age.

### Results and discussion

#### Desirability and purchase likelihood

Consistent with our previous findings, ANOVA results revealed significant main effects of presentation format on the desirability of the lamb shawarma (M_Control_ = 4.64 versus M_AR_ = 5.15; *F*(1,106) = 4.52, *p* = 0.04, η_p_^2^ = 0.04) and purchase likelihood (M_Control_ = 4.54 versus M_AR_ = 5.50; *F*(1,106) = 8.82, *p* < 0.01, η_p_^2^ = 0.08). These results add support for H1, in that participants who viewed the lamb shawarma in the AR condition rated both the desirability of the dish and their purchase likelihood of the dish as significantly higher than those who viewed the same lamb shawarma in the control (non-superimposed) condition.

As desirability and purchase likelihood are arguably related constructs (see Fuchs et al., 2015; Szocs et al., 2022) and were highly correlated (r = 0.76; *p* < 0.001), we conducted a factor analysis which demonstrated that all items loaded onto the same factor (the only factor with an eigenvalue greater than 1, accounting for 78% of the variation). Accordingly, we combined all 4 items into one aggregate product evaluation scale (*α* = 0.90) to streamline the reporting of the mediation analyses (described below; however we report all results using the subscales in Web Appendix G for completeness). As expected, ANOVA results indicated a significant effect of presentation format on this aggregated product evaluation measure (M_Control_ = 4.61 versus M_AR_ = 5.24; *F*(1,106) = 6.39, *p* = 0.01, η_p_^2^ = 0.06).

#### Personal relevance and mental simulation

To begin testing our proposed processes explaining the positive effect of AR presentation on product evaluations, we ran an ANOVA with presentation format as the predictor and both mental simulation and personal relevance as dependent variables. ANOVA results revealed a significant positive effect of presentation format on personal relevance (M_Control_ = 4.63 versus M_AR_ = 5.55; *F*(1,106) = 12.07, *p* = 0.001, η_p_^2^ = 0.10), where participants in the AR (vs. control) condition perceived the lamb shawarma to be more personally relevant to them. Similarly, ANOVA results revealed a significant positive effect of AR format on the degree to which participants engaged in mental simulation (M_Control_ = 4.59 versus M_AR_ = 5.14; *F*(1,106) = 5.11, *p* = 0.03, η_p_^2^ = 0.05), replicating the initial finding in the previous study.

Next, to test H2 and replicate the mediation results from the previous study, we ran a mediation analysis to determine whether the positive effect of presentation format on product evaluation could be explained through an increase in mental simulation. Mediation results (PROCESS Model 4, Hayes, 2018; with 10,000 resamples) with presentation format as the predictor variable, product evaluation as the dependent variable, and mental simulation as the mediator revealed a significant indirect effect (indirect effect = 0.3724, 95% CI: 0.0511 to 0.7420). Following the successful mediation through mental simulation, we next ran a sequential mediation analysis in order to test H3, the role of personal relevance as the precursory mechanism explaining the positive effect of AR presentation on mental simulation. Sequential mediation results (PROCESS Model 6, Hayes, 2018; with 10,000 resamples) with presentation format as the predictor variable, product evaluation as the dependent variable, and personal relevance followed by mental simulation as the sequential mediators revealed a significant indirect effect (indirect effect = 0.1636, 95% CI: 0.0440 to 0.3361),^10^ thus supporting H3 and the model proposed in Fig. 1. In other words, these results suggest that presenting the lamb shawarma in AR (vs. a non-super imposed format) led participants to deem it as more personally relevant, facilitating their mental simulation of consuming it, which ultimately improved their product evaluation.

#### Alternative process measures

To begin systematically assessing the potential alternative process measures, we first ran a series of ANOVAs with presentation format as the predictor and each potential process measure as a dependent variable. The full results are presented in Table 2.

**Table 2:** Study 3: Effect of AR presentation on potential process measures

DV	Mean		*F*	*p*	η_p_^2^
	Control	AR	(1, 106)		
Presence	-0.2*	0.2*	7.91	0.006	0.07
Attention to Background	1.57	1.93	3.03	0.09	0.03
Enjoyment of Experience	5.79	6.19	3.52	0.06	0.03
Fluency	5.41	5.72	1.68	0.20	0.02
Psychological Ownership	3.42	3.67	0.68	0.41	0.01
Spatial Distance	4.81	4.87	0.06	0.81	0.00
Temporal Distance	4.56	4.76	0.76**	0.38	0.01
Realism	5.96	5.98	0.01	0.91	0.00
Mood	5.84	5.84	0.00	1.00	0.00
					*Z-score
					**F(1,104)

ANOVA results revealed that of the nine potential alternative process explanations, presentation format exerted a significant positive effect on only one of the variables—presence (M_Control_ = -0.2* versus M_AR_ = 0.2* (*Z-scores); *F*(1,106) = 7.91, *p* = 0.006, η_p_^2^ = 0.07), suggesting that participants in the AR condition perceived the lamb shawarma to be more present in their peripersonal space than those in the control condition. In addition, results revealed marginally significant effects of presentation format on attention to the background (M_Control_ = 1.57 versus M_AR_ = 1.93; *F*(1,106) = 3.03, *p* = 0.09, η_p_^2^ = 0.03) and overall enjoyment of the experience (M_Control_ = 5.79 versus M_AR_ = 6.19; *F*(1,106) = 3.52, *p* = 0.06, η_p_^2^ = 0.03), in that participants in the AR condition reported paying more attention to the background and reported enjoying the experience more than those in the control condition. However, ANOVA results revealed no significant effects of presentation format on the remaining process measures, including fluency, psychological ownership, spatial and temporal distance, mood, or realism.

To test the efficacy of the three significant or marginally significant process measures, we then ran a parallel multiple mediation model with 10,000 resamples (Model 4, Hayes, 2018) including presentation format as the predictor (coded as control = 0 and AR = 1), product evaluation as the dependent variable, and presence, attention to background, and enjoyment of the experience as mediators. Mediation results did not reveal a significant indirect effect of presentation format on product evaluation through any of the three potential processes (with 95% confidence intervals straddling 0 for each measure). Therefore, we are able to reasonably rule out presence, attention to the background, and enjoyment of the experience as alternative process explanations, confirming the sequential mediation of personal relevance and mental simulation in H3 as the primary process explaining the positive effect of AR presentation on product evaluations.

#### Ancillary analysis

As in Study 1, to examine whether novelty might explain the positive effect of AR presentation on product evaluation, we test whether participants’ attitudinal response to presentation format differed according to their familiarity with AR technology. Once again, AR familiarity did not differ across the two presentation conditions (M_Control_ = 4.67 versus M_AR_ = 4.52; *F*(1, 106) = 0.217, *p* = 0.64, η_p_^2^ = 0.00). Importantly, neither the main effect of AR familiarity nor the interaction between presentation format and AR familiarity were significant predictors of product evaluation (both *p’s* > 0.59). This reinforces the findings in Study 1, suggesting that it is unlikely the positive attitudinal responses to foods presented in AR can be explained by the novelty of this technology.^11^

In sum, results of this laboratory experiment corroborate the findings of Studies 1 and 2 by demonstrating, in a controlled setting, that the real-time superimposition afforded by AR presentation increases both the desirability and purchase likelihood of depicted foods. Importantly, while this study replicated the mediational role of mental simulation, we were also able to identify personal relevance as the anteceding mediator in the process. In other words, superimposing a food onto a consumer’s real-time peripersonal space (via AR) leads consumers to deem it as more personally relevant, which facilitates their mental simulation of consuming it, and accordingly improves their evaluation of the food. While our results also demonstrate significant or marginally significant positive effects of AR presentation on other potential mechanisms (e.g., presence, attention to background, and enjoyment of the experience), they are unable to explain the effect of AR presentation on product evaluation.

## Study 4: Replicating the psychological process and extension to less desirable food category

Results from the previous three studies collectively established the positive effect of AR presentation on product evaluations, while supporting the sequential role of personal relevance and mental simulation as the underlying process explaining the observed effect. Aside from replicating the findings of Studies 1, 2 and 3, Study 4 served three additional purposes. First, the previous three studies utilized a mobile tablet (i.e., an iPad) to view both presentation formats (thus contributing theoretically to behavioral research in marketing that explores how technological features in mobile devices can systematically influence the way consumers process information and behave). However, given that consumer ownership and usage of mobile phones is significantly higher than that of tablets (Enge, 2021), we wished to extend the generalizability and robustness of our findings by examining the effect of AR presentation executed via a smartphone devices. Therefore, participants in all conditions of this study viewed the depicted food item on smartphone, allowing us to determine whether the results hold and replicate on a significantly smaller digital screen. Second, while Studies 1 and 2 focused on indulgent desserts, and Study 3 focused on a non-indulgent food item, Study 4 explicitly included a manipulation of food desirability, to examine whether the effects would continue to hold for relatively undesirable food items. Previous research (Labroo & Nielsen, 2010) has found that mental simulation can improve consumer attitudes towards not only desirable stimuli, but toward neutral and undesirable stimuli as well. For example, Labroo and Nielsen (2010) found that participants who were instructed to mentally simulate approaching an undesirable item (curried grasshoppers) reported significantly improved evaluations and willingness to pay compared to participants who were not instructed to mentally simulate approach.

Finally, our previous studies (and indeed, most research exploring mental simulation) treated mental simulation as a unidimensional construct. However, as theorized earlier, we expect the mental simulation facilitated through AR presentation should likely be driven by a process-oriented mindset rather than an outcome-oriented one, as the superimposition facilitated by AR provides users with the opportunity to imagine “engaging” with, or consuming, the food item. Notably, the mental simulation scale we used in the previous studies was comprised of items describing the consumption process (e.g., imagining eating) as opposed to a focus on any outcome per se. However, to more rigorously assess the process-oriented versus outcome-oriented distinction, we include additional items from previous literature to distinctively and separately measure both process-oriented and outcome-oriented simulation.

### Design and procedure

One hundred and seventy-three volunteers (44% female, 0% Non-binary/other; M_Age_ = 29.87, SD = 7.00) from a university setting participated in this experiment in exchange for monetary compensation. This study employed a 2 (presentation format: control vs. AR) × 2 (food item: undesirable vs. desirable) between-subjects design. Presentation format was manipulated as in Study 2 and 3 (keeping scale, resolution, dimensionality, and interactivity constant): the control condition featured the 3D food item over a static blank background, while the AR condition superimposed the 3D food item onto the viewer’s real-time environment. To manipulate food-item desirability, participants either viewed fermented trout (determined as “undesirable” in the pretest reported in Web Appendix H), or parmesan fries (determined as “desirable” in the pretest reported in Web Appendix H). Images of the stimuli are presented in Appendix G.

Once participants arrived at the lab, they scanned a QR code with their personal mobile phone to access a survey that included the consent form and survey questions. Participants were told that the purpose of this study was to view and evaluate a food item. Next, participants were provided with a Samsung S21 smartphone, and were randomly assigned to view either the undesirable food item (i.e., fermented trout), or the desirable food item (i.e., parmesan fries), in either the control or AR format. Prior to viewing the randomly assigned item, but after being told what food item they would be viewing, participants were asked to indicate their previous experience with the assigned food (“I have eaten fermented trout/parmesan fries before*,”* with the options, “yes,” “no,” or “unsure”).

Participants responded to survey questions on their mobile phones while continuing to view the food item on the mobile phone provided (should they wish to). Our dependent variable of interest in this study was product evaluation (using the 4-item scale from Study 3; *α* = 0.96). In an attempt to measure and replicate the sequential mediation observed in Study 3, participants responded to the same personal relevance (*r* = 0.72), and mental simulation (*α* = 0.91) measures that were employed in Study 3. To further investigate the type of mental simulation participants might engage in, they were also asked to respond to a 2-item index measuring process-oriented simulation (2-item measure adapted from Castaño et al., 2008; e.g., “I thought about how I would eat this food item,” “I thought about the process of eating this food item”; *r* = 0.72), and a 2-item index measuring outcome-oriented simulation (2-item measure adapted from Castaño et al., 2008; e.g., “I thought about why I would eat this food item,” “I thought about the benefits I would gain from eating this food item”; *r* = 0.34). Participants indicated the realism of the stimuli (*α* = 0.85), their overall mood (*r* = 0.74), and their familiarity with AR Technology, all measured as in Study 3. Finally, they indicated their gender and age (see Web Appendix I for complete list of measures).

### Results and discussion

#### Product evaluation

**Fig. 2:**
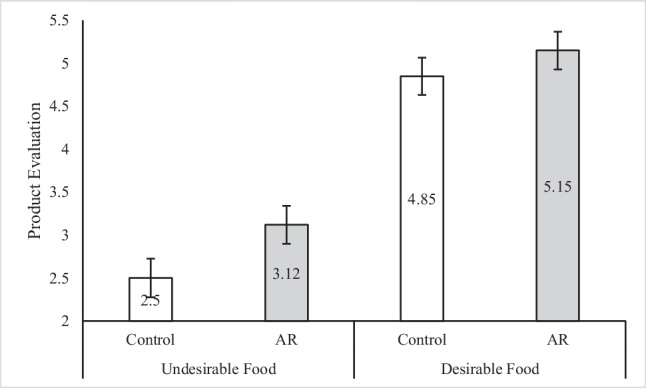
Study 4: interactive effect of presentation format and food-item desirability on product evaluation

Upon entering the lab, one participant mentioned they were familiar with the research as one of the researchers was their instructors. We accordingly excluded this participant from analysis, in addition to one participant whose evaluation of the undesirable food item was more than two standard deviations above the mean (i.e., an outlier; Porath et al., 2010), resulting in one hundred and seventy-one observations for analysis (we include the results of the analysis while retaining these participants in Web Appendix J). A 2 × 2 ANOVA revealed significant main effects of presentation format (*F*(1, 167) = 4.39; *p* = 0.04, η_p_^2^ = 0.03) and food item (*F*(1, 167) = 98.21; *p* < 0.001, η_p_^2^ = 0.37) on overall product evaluation. As was the case in our previous studies, product evaluation was higher in the AR condition (M_AR_ = 4.14) compared to product evaluation in the control condition (M_Control_ = 3.67). As to be expected, product evaluation was also higher in the Desirable (i.e., parmesan fries) condition (M_Desirable_ = 5.00), compared to product evaluation in the Undesirable (i.e., fermented trout) condition (M_Undesirable_ = 2.81). The interactive effect was not significant (*F*(3, 167) = 0.50, *p* = 0.48), suggesting that AR presentation boosted desirability ratings for both products in a similar manner. An illustration of means is presented in Fig. 2.

#### Personal relevance

2 × 2 ANOVA results for personal relevance follow the same pattern observed on product evaluation, indicating a significant main effect of presentation format (*F*(1, 167) = 8.93; *p* < 0.01, η_p_^2^ = 0.05) on personal relevance, suggesting that participants in the AR condition (M_AR_ = 4.32) perceived the food items to be more personally relevant to them, compared to participants in the control condition (M_Control_ = 3.58). Similarly, ANOVA results revealed a significant main effect of food item (*F*(1, 167) = 41.31; *p* < 0.001, η_p_^2^ = 0.20) on personal relevance, again suggesting that participants who viewed the desirable food item (M_Desirable_ = 4.75) perceived it to be more personally relevant than those who viewed the undesirable food item (M_Undesirable_ = 3.15). The interactive effect between presentation format and food item was not significant (*F*(3, 167) = 1.35; *p* = 0.25, η_p_^2^ = 0.01).

#### Mental simulation

2 × 2 ANOVA results on mental simulation followed the same pattern as product evaluation and personal relevance. ANOVA results revealed significant main effect of presentation format (*F*(1, 167) = 4.24; *p* = 0.04, η_p_^2^ = 0.03) on our 6-item measure of mental simulation, suggesting that participants in the AR condition (M_AR_ = 4.78) reported significantly higher mental simulation of consuming the food item compared to participants in the control condition (M_Control_ = 4.33). Similarly, ANOVA results revealed a significant main effect of food item (*F*(1, 167) = 14.97; *p* < 0.001, η_p_^2^ = 0.08) on mental simulation, suggesting that participants who viewed the desirable food item (M_Desirable_ = 4.98) reported significantly higher mental simulation of consuming the depicted food compared to those who viewed the undesirable food item (M_Undesirable_ – 4.13). Once again, the interactive effect was not significant (*F*(3, 167) = 1.61, *p* = 0.21, η_p_^2^ = 0.01). We next ran a sequential mediation analysis to test H3 and replicate the mediation observed in Study 3 to explore whether the effect of presentation format on product evaluation could be explained through an increase in personal relevance, followed by an increase in mental simulation. Specifically, we conducted a sequential mediation analysis (PROCESS Model 6, Hayes, 2018; using 10,000 resamples) with presentation format as the predictor (coded as control = 0, AR = 1), product evaluation as the dependent variable, personal relevance followed by mental simulation as the sequential mediators, and food item as a covariate. Results revealed a significant indirect effect of presentation format on product evaluation through personal relevance and mental simulation (indirect effect = 0.1272, 95% CI: 0.0379 to 0.2410), supporting H3 and replicating the results of Study 3.

To specifically determine what type of mental simulation (process-oriented versus outcome-oriented) might explain the observed results, we first conducted a factor analysis with the 6 general mental simulation items, the 2 process-oriented simulation items, and the 2 outcome-oriented simulation items (see Web Appendix K for details). Results indicated that the two process-oriented items loaded onto the same factor as all 6 mental simulation items used in our original mental simulation scale (the only factor with an eigenvalue greater than 1.3, accounting for 51% of the variation). Accordingly, we combined all 8 items into one aggregate “process-oriented mental simulation” scale (*α* = 0.90). Sequential mediation analysis results using this aggregated scale mirror the results reported above (see Web Appendix L for details of the analysis), providing further support for the role of both personal relevance and mental simulation as the underlying processes explaining the observed effect of AR presentation on product evaluation, and identifying process-oriented simulation as the specific type of mental simulation explaining the effect.^12^

In sum, results of this laboratory experiment further supported the findings of Studies 1, 2, and 3 by demonstrating that the real-time superimposition afforded by AR presentation increases the perceived personal relevance of the depicted food item, which increases the process-oriented mental simulation participants experience of consuming the depicted food item, ultimately increasing the overall evaluation of the product. Importantly, while this study replicated the sequential mediational role of personal relevance followed by mental simulation, we were also able to uncover the specific type of mental simulation observed: process-oriented simulation. In other words, it appears as though the increased personal relevance resulting from the superimposition of a food item onto a consumer’s real-time peripersonal space (via AR) leads the consumer to simulate, mentally, the process of interacting with and consuming the depicted food item (compared to imaging the outcome resulting from eating the food item), ultimately improving their evaluation of the food.

Importantly, this study allowed us to explore whether the positive effect of AR presentation works only for desirable food items (as tested in the previous 3 studies), or if AR presentation could also improve evaluations of undesirable food items. Consistent with previous literature (Labroo & Nielsen, 2010), findings from this study demonstrate that the positive effect of AR presentation does not only manifest for desirable food items (e.g., parmesan fries), but also extends to less-desirable food items (e.g., fermented trout).

Finally, Study 4 provided an opportunity to test whether the observed results from the previous three studies could be replicated on a smaller, yet more accessible, mobile device (i.e., smartphones). While we did not explicitly manipulate and compare device form (i.e., iPad vs. smartphone) in this study, the positive effect of AR presentation, and the replication of the sequential process from the previous study, add to the robustness of our investigation extend the generalizability and relevance of our investigation.

## General discussion

Industry voices have both heralded AR technology for its potential, and criticized it for being over-hyped (Sullivan, 2021, WIRED, 2021). Motivated in part by these polarized perspectives, our research empirically examines how and why this increasingly pervasive technology might influence consumers’ judgements and behaviors in the marketplace. Specifically, we find that because AR visually superimposes objects onto a consumer’s real-time environment, it leads consumers to percieve depicted foods to be more personally relevant, increasing mental simulation and improving downstream attitudes and behaviors. Across two field studies and two laboratory experiments, we collectivley show that presenting foods in AR (versus either an externally valid 2D static format or a more conservative dynamic 3D format) can ultimately increase its perceived desirability and increase consumers’ purchase likelihood. Importantly, we find the positive effect of AR presentation holds across both large (e.g., tablets; Studies 1–3) and small (e.g., smartphones; Study 4) mobile devices, regardless of whether the food is immediately available for consumption (Studies 1 and 2) or not (Studies 3 and 4), and in the context of indulgent (i.e., dessert in Studies 1 and 2), non-indulgent (Study 3), and both desirable and undesirable (Study 4) food categories.

### Theoretical contributions

The current work addresses recent calls to explore how AR affects sensory perceptions, decision-making, attitude formation, and pre/post purchase behavior and evaluations (Tan et al., 2022) and how new immersive consumer technologies—including AR—can affect consumers’ mental simulation and imagery generation (Ceylan et al., 2022). While existing literature on AR has advanced our understanding of what optimal settings within AR applications might look like, we empirically isolate what we consider the core, unique facet of AR presentation: real-time superimposition of visual objects in one’s environment. Specifically, we demonstrate that due to its ability to visually superimpose products onto a consumer’s real-time environment, AR presentation uniquely able to generate perceived of personal relevance and mental simulation *above and beyond* visual stimuli that is not superimposed, contributing to this literature by both identifying and measuring the psychological processes driving the positive effect on desirability and purchase likelihood.

We also add to a growing body of behavioral research in marketing that explores how technological features in mobile devices can systematically influence the way consumers process information and behave. While previous scholars have examined the touchscreen (Brasel & Gips, 2014; Shen et al., 2016), haptic feedback (Hadi & Valenzuela, 2020) and photo-taking (Diehl et al., 2016) functionality of mobile devices, the current research demonstrates that AR superimposition using a mobile device’s camera can also alter consumers’ perceptions and influence real-world behaviors.

As mentioned above, our documentation of the positive effect of AR presentation on consumer responses to foods appears to be relatively robust (generalizable across different device sizes, food categories and consumption contexts). However, given our delineation of the underlying process, we can make some logically informed inferences about the generalizability of our findings apply beyond the food domain. Namely, since our research demonstrates mental simulation is one of the critical mechanisms through which AR exerts its effects on consumer responses, positive consumer responses should theoretically manifest in other contexts where mental simulation is considered advantageous. Given that previous research suggests mental simulation is most beneficial for highly sensorial/hedonic (as opposed to utilitarian) product attributes (MacInnis & Price, 1987) and our finding that the specific mental simulation at work is likely process-focused (as opposed to outcome-focused) in nature, AR presentation should theoretically improve responses when consumers are focused on hedonic attributes that arise while using a product. For example, if a consumer is shopping for a kitchen appliance, AR presentation might improve the product’s desirability if the consumer is focused on how fun it would be to use the appliance, as opposed to being focused on a utilitarian outcome (e.g., the result of using the appliance). We expand on these potential extensions in the “future research” section further below.

### Practical implications

As mentioned in the introduction to this paper, some food and beverages establishments brands have begun experimenting with AR applications, but it is far from being an ubiquitous practice. Our findings suggest that offering consumers the ability to view products in an AR format may be a worthwhile investment, and this can be implemented in a number of ways. In the context of the food/restaurant industry that we have focused on, many dining establishments (e.g., restaurants, cafeterias, bars) already offer patrons digital menus via handheld tablets (Anindita, 2018). These digital menus can easily be upgraded with AR-enabled renderings of the menu items, allowing patrons to visually preview dish on their table before placing an order (similar to the procedure we used in Studies 1 and 2). Catering firms and bakeries that provide custom offerings (e.g., personalized cakes) can use AR technology to facilitate potential customers’ ability to visualize what yet-to-be-created products will look like, before going through any irreversible production process. Our research suggests that such efforts should make the viewed food items more desirable and lead to increased purchase likelihood. Further, given our finding that AR previewing improves consumers’ evaluation of the consumption experience itself, it is likely to increase customer satisfaction (itself a critical determinant of marketplace behaviors such as repurchase, recommendation, and willingness to pay; Anderson & Sullivan, 1993; Homburg et al., 2005) and result in fewer returns and complaints.

Additionally, because AR technology simply requires a camera-enabled mobile device, these implications carry over to consumers’ at-home viewing of foods. This has become an increasingly relevant given that online food ordering (e.g., Uber Eats, Seamless, Deliveroo) and online grocery shopping have both been on the rise (Kats 2019, Littman 21,019), and even more so since the Covid-19 pandemic (Venkataramakrishnan 2020). AR technology can give consumers the opportunity to view food offerings in their own homes, on their own dining room tables, before placing any orders. In fact, given that one of the underlying mechanisms we uncover is personal relevance, AR presentation might prove even more beneficial in such circumstances. That is, superimposing the featured products not only in a user’s peri-personal perimeter, but also within the user’s intimate household settings might exaggerate the effects on perceived personal relevance, and accordingly amplify the effects on mental simulation and downstream variables accordingly.

In addition, the camera-enabled nature of mobile devices means these implications can also extend to mobile marketing efforts. Brand managers can use AR tools embedded within Snapchat, Instagram, or TikTok filters to present foods in consumers real-time environments and can even enable transactions through those channels (as Domino’s pizza did; Swant, 2018). These opportunities will likely become more common in the future given the developments of AR glasses by the aforementioned tech giants (Swanner 2019).

Aside from the implications for practitioners, it is worthwhile to consider what the current research might mean from a consumer welfare perspective. At first blush, the notion that AR presentation increases food desirability and purchase may suggest that the practice is potentially detrimental for consumer well-being, particularly given the already-widespread tendency for consumers to overeat and the related obesity epidemic (Gao et al., 2022; Scott et al., 2008). Indeed, several health organizations and scholars have attributed the increasingly obesogenic environment to the ubiquitous and compelling nature of food media (Bublitz et al., 2010). However, the implications of our findings are likely more nuanced than such a conclusion would suggest. In particular, the results show that AR presentation does not only increase the desirability of indulgent and unhealthy foods (e.g., the desserts in Studies 1 and 2), but also functioned to increase the desirability of a non-indulgent food (the lamb shawarma in Study 3, as per pretest results in Web Appendix D) and even an otherwise undesirable food item (e.g., the fermented trout in Study 4, as per pretest results). This suggests that AR presentation may very well increase the desirability and purchase of healthy foods as well, which could, in the right contexts, have a beneficial influence on consumer health and well-being. Furthermore, AR presentation may also be a means of encouraging consumers to more readily imagine consuming foods that they are less familiar with (by creating the perception that they are more personally relevant) and could thus possibly provide benefits related to epicurean exploration and learning. This notion represents an interesting route for further investigation, more of which we discuss next.

### Future research

The burgeoning nature of AR technology and its expanding marketplace applications pave several exciting avenues for potential future research, both within the food domain and beyond. For example, while the current work focused on how AR technology can be used during consumers’ previewing and ordering of foods, it would also be interesting to examine how AR can also be applied during consumers’ consumption of foods and beverages. For example, at Sublimotion, the world’s most expensive restaurant, diners wear headsets and are treated to a 15-course gastronomic show combining gourmet cuisine with AR intended to, “play with emotions, the senses, the set, the aromas, and the taste to be able to create absolutely unique experiences for each scene [course],” (Strause, 2015). In the beverage space, Australian wine company *19 Crimes* created an AR app allowing consumers to bring the wine bottle’s label to life through their mobile devices: criminals featured on each bottle become animated and tell their story, enriching the drinking experience by simultaneously engaging both the mind and taste buds (Stone, 2017). It is likely that such applications can significantly transform consumers’ consumption experiences, turning them into highly interactive and experiential episodes.

It is also worth reiterating that this investigation leveraged an AR application where the device’s rear-facing camera (i.e., the camera on the back of the phone, on the side opposite from the screen) projects visual content into the user’s current space. As outlined earlier, other AR applications utilize the device’s front-facing camera (i.e., the camera on the screen-side of the phone, sometimes referred to as the “selfie” camera) to superimpose visual content (e.g., clothing, accessories, or makeup) onto the users themselves (Hilken et al., 2017; Poushneh & Vasquez-Parraga, 2017; Smink et al., 2019; Tan et al., 2022; Yim et al., 2017). It is interesting to consider cases where such applications might visually “transform” the user themself in an effort to show the consequences of food consumption (e.g., a recent Instagram filter shows users’ faces getting fuller if they repeatedly indicate a preference for unhealthy foods). It seems plausible that outcome-oriented mental simulation might become more relevant than process-oriented mental simulation in such cases, and it would be interesting to explore whether such presentations can motivate healthier eating behavior and/or how they might modulate users’ self-image perceptions. This represents a potentially fruitful area for further investigation.

While applications in the food and beverage domain provided a theoretically and externally valid area to examine the effect of AR’s real-time superimposition on consumer responses, it could be interesting and worthwhile to examine how our uncovered effects and mechanisms may or may not apply across other product categories. As alluded to earlier, this might involve systematic investigations into whether the effect and process we found in the current work differentially apply to other hedonic versus utilitarian categories and/or contexts. Further, while we found one dominant sequential mechanism explaining the effect in our studies (personal relevance and mental simulation, respectively), the effect is likely driven by multiple processes (including perceived presence, attention to the background, and enjoyment of the experience), and these variables may become more or less relevant in alternative contexts that future researchers may wish to explore.

Shifting further afield, it is compelling to consider how future research might move beyond products altogether and explore how AR presentation influences consumer responses to the real-time superimposition of other human beings. For example, Google recently created AR “stickers” of the Grammy nominated rapper Childish Gambino, giving users the ability to see a visual depiction of the artist performing in their current environment (Holt, 2019). It is interesting to consider whether and how such applications might influence consumer connections to the individuals who are visually superimposed into their spaces, and how that may or may not extend to the companies and brands sponsoring the content.

While mobile AR is here now (enabled on the billions of Android and Apple smartphones worldwide), the imminent fusion of AR technology into wearable devices (i.e., glasses) will make it an even more permeating phenomenon. One tech executive described this future by saying, “The world is about to be painted with data,” (Fink, 2018). In such an analogy, marketers may very well be holding the paintbrushes, and we hope to see more research exploring how AR can best be used to enhance the customer experiences and marketing outcomes accordingly.

### Electronic supplementary material

Below is the link to the electronic supplementary material.

Supplementary file1 (DOCX 938 KB)

## Appendix C: Presentation format manipulation in Study 1

*Control Condition*

*AR Condition*

## Appendix D: Dessert options in Study 2

*Treacle Tart*

*Summer Berries*

*Brownie*

## Appendix E: Presentation format manipulation in Study 2

*Control Condition*

*AR Condition*

## Appendix F: Presentation format of lamb shawarma in Study 3

*Control Condition*

*AR Condition*

## Appendix G: Study 4 stimuli

Desirable: Parmesan Fries

Undesirable: Fermented Trout

## Footnotes

1 While we focus this research within the food domain for aforementioned reasons, it is worth noting that AR is actively being used in a variety of different domains beyond food and beverages, including the entertainment industry, education, art, commerce, and more. See Web Appendix A for additional marketplace examples.

2 At first blush, it may seem as though AR superimposition was manipulated in work by Heller and colleagues (2019a). While the majority of their studies focus on imagery transformation (i.e., the ability to manually manipulate or transform the content with one’s fingers), in one of the five studies they also manipulate imagery generation (low vs. high). In the low AR imagery generation configuration, participants received augmented information such as dessert ingredients and price, while in the high AR imagery generation configuration, participants viewed digitized replicas of each dessert on their table. This study significantly differs from our investigation in two main ways: it manipulated the content of augmented information (whereas we hold content constant), and it focused on word of mouth effects as the central dependent variable (whereas we focus on desirability and purchase).

3 We received ethical approval from an institutional review board for all these experiments before commencing any of the data collection.

4 All diners at the restaurant during dinner shift (6 pm – 10:30 pm) who ordered a “main dish” were approached (108 in total). Sample sizes our subsequent studies were similarly determined by logistical constraints on data collection.

5 As a robustness check, because individuals were nested in tables, we also ran a multilevel binary logistic regression that modelled table as a random factor to account for table-specific effects. Results of a model including presentation format as a predictor, dessert purchase as the dependent variable, and number of people at each table and the average money spent per person as covariates confirmed a significant effect of AR presentation (*F*(1, 97) = 5.41, *p* = .02).

6 Previous research has produced mixed findings regarding how group size influences food consumption (Herman 2015). Some research suggests that consumers eat less food when they dine with strangers (as opposed to with close friends; Clendenen, Herman and Polivy 1994). It is possible that in our study, smaller groups were comprised of close friends while larger groups were comprised of more distant acquaintances or work colleagues, however, it is difficult to engage in such extrapolation as we do not have data assessing such social considerations.

7 While we adopted the measure of AR familiarity in all of our studies from previous literature, we acknowledge that one limitation is that it is not domain-specific (e.g., it refers to overall AR familiarity as opposed to AR in the context of food presentation).

8 We also ran a multilevel linear regression that modelled table as a random factor to account for table-specific effects. Results of a model including presentation format as a predictor, average dessert desirability as the dependent variable, and number of people at each table and the average money spent per person as covariates confirmed a significant effect of AR presentation (*F*(1, 97) = 17.02, *p* < .001).

9 While we had no a priori predictions about the effect of AR presentation on post-consumption enjoyment, we tested an exploratory mediation analysis (model 4, Hayes 2018) with 10,000 resamples) to see whether mental simulation could also explain the increased post-consumption enjoyment induced by AR. Results did not demonstrate a significant indirect effect (indirect effect = -.0386, 95% CI: -.1509 to .0465). We discuss other potential mechanism in the general discussion.

10 While the reverse sequential mediation pathway (i.e., mental simulation followed by personal relevance) also produced a significant indirect effect (indirect effect = .0734, 95% CI: .0070 to .1926), the larger effect size of the predicted sequential mediation suggests to us that it represents a more likely process explanation than the reverse. This path is also theoretically supported by extant research (Ülkümen and Thomas 2013), demonstrating that high (vs. low) levels of personal relevance increases consumers’ tendency to spontaneously engage in mental simulation.

11 We were also interested in exploring whether participants’ prior experience with the featured food item might moderate their response to presentation format. An ANOVA with presentation format and prior experience as predictors and product evaluation as the dependent variable revealed a marginally significant interaction between presentation format and prior experience (F(1,104) = 3.45, *p* = .07, η_p_^2^ = .03). An analysis of contrasts revealed that the positive effect of AR format on product evaluation was significant for people who had not eaten lamb shawarma before (F(1,104) = 9.08, p < .01, ηp2 = .08), but was not significant for those who had eaten lamb shawarma before (F(1,104) = .27, p = .60). while this finding was intriguing, prior experience did not emerge as a significant moderator in our following study. The inconsistency, paired with the fact that many of product categories in Studies 1, 2, and 4 were indeed familiar (e.g., brownies, french fries) and still showed a positive response to AR presentation, led us to conclude that the moderating effect of prior experience in the current may have been spurious.

12 We also tested the reverse order of mediators (i.e., process-oriented mental simulation followed by personal relevance) but the indirect effect was not significant (95% CI: [-.0005, .2225]). It is worth noting that conceptually, all of the items from the original mental simulation scale (which works as a mediator in Studies 2–4) involve the consumption process (e.g., imagining eating) as opposed to a focus on any outcome per se. However, to rigorously examine whether the specific type of mental simulation at work is process-oriented simulation, we ran a number of robustness checks (see details in Web Appendix M). Results indicate that the two-item process-oriented mental simulation index (using only the two items from previous literature) also works in our proposed model. Meanwhile, the two-item outcome-oriented mental simulation index did not emerge as a significant mediator in our model.
