# Supplementary material for: From tablet to table: How augmented reality influences food desirability
Source: J Acad Mark Sci. 2022 Dec 27;51(3):503–29. doi: 10.1007/s11747-022-00919-x (PMC9792938; doi:10.1007/s11747-022-00919-x)
Supplement: Supplementary file 1 — Supplementary file1 (DOCX 938 KB) [file 11747_2022_919_MOESM1_ESM.docx]

**Web Appendix a**

**APPLICATIONS OF AR IN CONSUMER EXPERIENCE DOMAINS**

| **industry** | **case*** |
| --- | --- |
| Art | [**Apple ART Walk**](https://www.apple.com/uk/newsroom/2019/07/apple-offers-new-augmented-reality-art-sessions/)  In 2019, Apple commissioned interactive AR art from six of the world’s premier contemporary artists to connect participants with public spaces in six international cities.  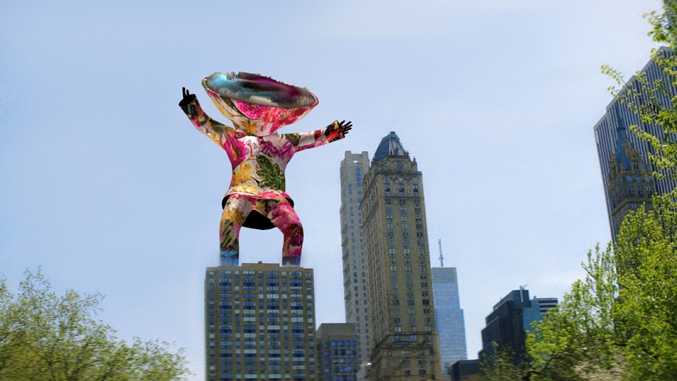 |
| Beauty | [**ModiFace**](https://www.loreal.com/en/beauty-science-and-technology/beauty-tech/discovering-modiface/)  ModiFace, acquired by L’Oréal in 2018, allows potential consumers to virtually “try-on” a variety of beauty products from foundation and lipstick to different hairstyles or colors.  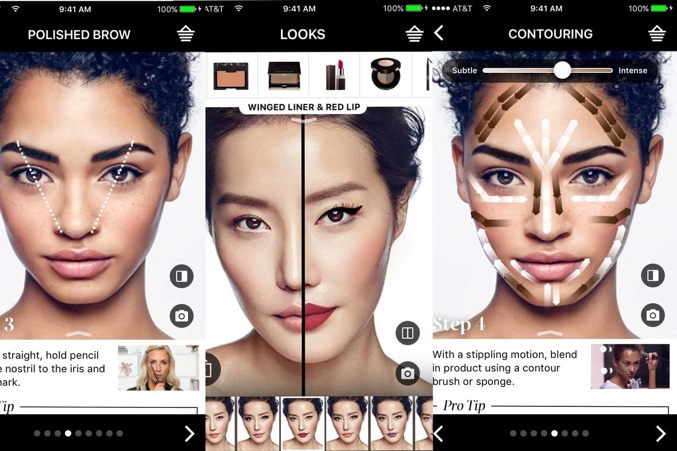 |
| Education | [**Google Arts and Culture**](https://artsandculture.google.com/)  Google has compiled over 1000+ unique (and free) AR & VR immersive learning experiences to enable students and teachers to explore some of the world’s most famous landmarks, museums, environments, and more, all from the comfort of their own home or classroom.  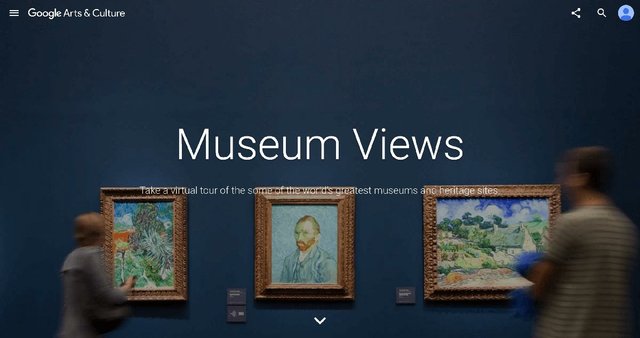 |
| Entertainment | [**British National Theatre Immersive Storytelling Studio**](https://www.nationaltheatre.org.uk/immersive)  The United Kingdom’s National Theatre has established a cutting-edge experimental studio to explore how VR, AR, 360º film and other immersive technologies can be used to enhance and broaden the audience experience of traditional live performances.  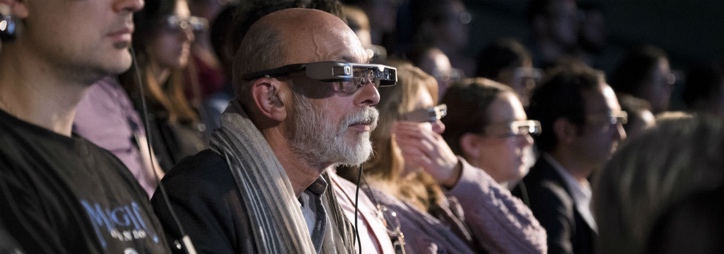 |
| Retail | [**ikea Place App**](https://www.ikea.com/gb/en/customer-service/mobile-apps/)  The IKEA Place App allows customers to visually superimpose life-sized IKEA furniture into their home prior to purchasing, allowing them to visualize how different items would look and fit within their home.  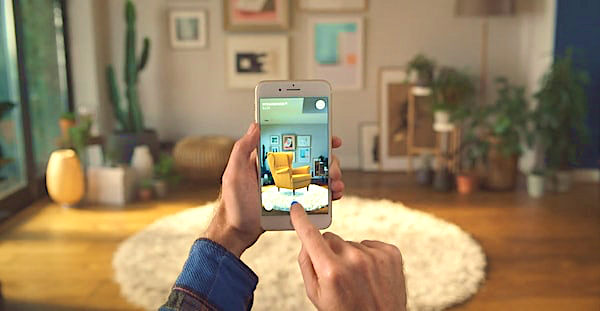 |

*click on link for more information on each

**WEB APPENDIX B**

**Study 1: Complete List of Measures**

1. *How Desirable Did You Find Each Dessert You Viewed? [7 Point Scale, 1=Very Undesirable, 7=Very Desirable]*
   1. *Crème Brûlée*
   2. *Stucky Toffee Pudding*
   3. *Strawberry Trifle*
   4. *Berry Pavlova*
   5. *Belgian Chocolate Truffle Cake*
   6. *Eton Mess*
2. *Did you order a dessert? [Yes, No]*
3. *If no, why did you not order dessert? [open response]*
4. *If yes, which dessert did you order? [open response]*
5. *Why did you choose the dessert you did? [open response]*
6. *Were you planning to get dessert even before seeing the menu? [Yes, No]*
7. *How familiar with Augmented Reality technology are you? [7 Point Scale, 1=Very Unfamiliar, 7=Very Familiar]*

**WEB APPENDIX C**

**Study 2: Complete List of Measures**

1. *Dessert Selected: Summer Berries OR Treacle Tart OR Chocolate Brownie*
2. *Please answer the following BEFORE RECEIVING YOUR CHOSEN DESSERT: [7 Point Scale, 1=Strongly Disagree, 7=Strongly Agree]*
   1. *I am craving the dessert I chose.*
   2. *When viewing the dessert, I could imagine myself eating it.*
   3. *I think I will enjoy the dessert I chose.*
   4. *I feel confident the dessert I chose will taste good.*
3. *Please answer the following AFTER EATING YOUR CHOSEN DESSERT: [7 Point Scale, 1=Strongly Disagree, 7=Strongly Agree]*
   1. *I enjoyed the dessert.*
   2. *I am happy I chose the dessert I did*

| **IV** | **Measure** | | **Mean** | | | ***F*** | | | ***p*** | **η_p_^2^** |
| --- | --- | --- | --- | --- | --- | --- | --- | --- | --- | --- |
|  |  | | **Control** | | **AR** | **(1, 127)** | | |  |  |
| **Pre-Consumption** | | | | | | | | | | |
| Condition | “I am craving the dessert I chose” | | 4.90 | | 5.46 | 8.402 | | | .004 | .062 |
| Condition | “I think I will enjoy the dessert I chose” | | 5.93 | | 5.94 | .004 | | | .950 | .000 |
| Condition | “I feel confident the dessert I chose will taste good” | | 5.66 | | 5.94 | 2.980 | | | .087 | .023 |
| Condition | “When viewing the dessert, I could imagine myself eating it” | | 5.49 | | 5.90 | 5.189 | | | .024 | .039 |
| **Post-Consumption** | | | | | | | | | | |
| Condition | “I enjoyed the dessert” | | 5.59 | | 6.03 | 4.024 | | | .047 | .031 |
| Condition | “I am happy I chose the dessert I did” | | 5.58 | | 6.08 | 3.985 | | | .048 | .030 |
| Condition | “The dessert looked the way it was pictured” | | 5.78 | | 6.24 | 3.985 | | | .048 | .030 |
| Condition | “The dessert tasted the way I imagined it would” | | 4.75 | | 5.68 | 12.632 | | | .001 | .090 |
|  | |  | |  | | |  |  |  |  |
|  | |  | |  | | |  |  |  |  |

- 1. *The dessert looked the way it was pictured.*
  2. *The dessert tasted the way I imagined it would.*

**WEB APPENDIX D**

**Study 3: Lamb Shawarma Pretest Results**

Fifty-one participants (41% Female, 59% Male, 0% nonbinary/other; M_Age_ = 41.90, SD = 12.35) recruited from Amazon Mechanical Turk participated in this pretest in exchange for monetary compensation. All participants viewed a static 2D picture of the Lamb Shawarma pictured in Appendix F and were asked to rate how indulgent they perceived the Lamb Shawarma to be on three bipolar items (1 = Unhealthy/High in Calories/Bad for You and 7 = Healthy/Low in Calories/Good for you; *α* = .90). Analysis results indicated that the Lamb Shawarma (M = 3.21, SD = .96) was considered to be neutral on this scale (neither indulgent nor healthy), as the mean was not significantly different from the mid-point (*t*(50) = 1.554, *p* = .13).

**WEB APPENDIX E**

**Study 3: COVID-19 Guidelines**

To ensure the health and safety of individuals who voluntarily agreed to participate in this Study, we followed strict COVID-19 guidelines set forth by both the Government and University. COVID-19 compliance guidelines are found below:

1. All participants were required to scan an ID card to access the building as part of a Track and Trace initiative. This also required participants to have their temperature checked prior to accessing the building to ensure they did not have a fever. Although participant identities were known for purposes of Track and Trace, their identity was not linked to their responses in this study in any way.
2. All participants were required to wear a face mask for the entire time they were in the building (this includes while waiting to begin the study, while completing the study, and after the study had ended).
3. In order to ensure 2 meters/6 feet between participants, a lecture theatre with a normal capacity of 84 people was converted into a Laboratory, with a maximum of 20 participants. Participants were spread throughout the room with seats blocked off to ensure proper distancing. See photo below representing the room setup.
4. Between each session all hard surfaces (door handles, desks, iPads, etc.) were professional cleaned. Hand sanitizer was also provided to participants before and after the session if they wished to use it.


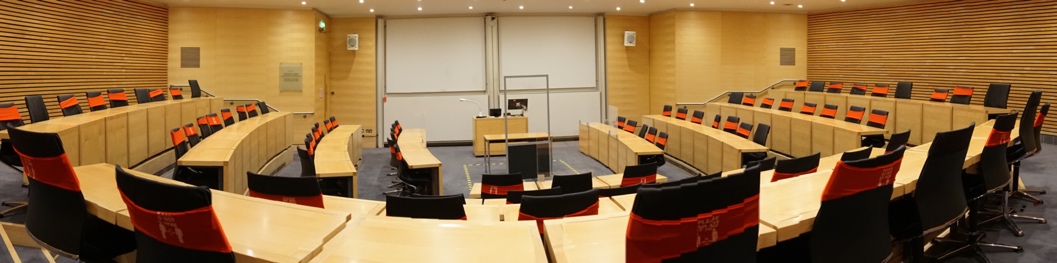


**WEB APPENDIX F**

**Study 3: Complete List of Measures**

- **Desire** *[see individual item for scale]*
  - *How desirable did you find the Lamb Shawarma to be? [7 Point Scale, 1=Extremely Undesirable, 7=Extremely Desirable]*
  - *How strong is your urge to eat this Lamb Shawarma? [7 Point Scale, 1=Extremely Weak, 7=Extremely Strong]*
  - *How strong are your cravings for this Lamb Shawarma? [7 Point Scale, 1=Extremely Weak, 7=Extremely Strong]*
- **Purchase Likelihood** *[7 Point Scale, 1=Extremely Unlikely, 7=Extremely Likely]*
  - *After viewing the Lamb Shawarma, how likely would you be to order it if it was offered on a menu?*
- **Dietary Restriction** *[Yes (please explain), No, Unsure]*
  - *I have a food allergy or dietary restriction which prevents me from consuming this food item.*
- **Mental Simulation** *[7 Point Scale, 1=Strongly Disagree, 7=Strongly Agree]*
  - *I could imagine myself eating the Lamb Shawarma displayed.*
  - *I could see myself eating the Lamb Shawarma displayed.*
  - *I thought about myself eating the Lamb Shawarma displayed.*
  - *The thought of myself eating the Lamb Shawarma felt real.*
  - *The thought of myself eating the Lamb Shawarma was very clear.*
  - *The thought of myself eating the Lamb Shawarma was very vivid.*
- **Presence** *[see individual item for scale]*
  - *The Lamb Shawarma felt like it was on the table in front of me. [7 Point Scale, 1=Strongly Disagree, 7=Strongly Agree]*
  - *I momentarily forgot the Lamb Shawarma was not real. [7 Point Scale, 1=Strongly Disagree, 7=Strongly Agree]*
  - *I perceived the Lamb Shawarma to be located: [5 Point Scale, 1=Entirely on my iPad, 5=Entirely on the table in front of me]*
- **Attention to Background** *[7 Point Scale, 1=Strongly Disagree, 7=Strongly Agree; reverse coded]*
  - *I paid more attention to the Lamb Shawarma than I did the background behind it.*
- **Attempted Touch** *[7 Point Scale, 1=Strongly Disagree, 7=Strongly Agree]*
  - *I felt like I wanted to reach out and touch the Lamb Shawarma.*
  - *I reached out to try to touch the Lamb Shawarma.*
- **Spatial Distance** *[see individual item for scale]*
  - *How close do you think the restaurant offering the Lamb Shawarma is located? [7 Point Scale, 1=Very Far, 7=Very Close]*
  - *How many miles away do you think the restaurant offering this Lamb Shawarma is located? [open response]*
- **Temporal Distance** *[see individual item for scale]*
  - *How quickly do you think the restaurant could deliver this Lamb Shawarma to you? [7 Point Scale, 1=Very Slow, 7=Very Fast]*
  - *How many minutes do you think it would take the restaurant to deliver this Lamb Shawarma to you? [open response]*
- **Fluency** *[7 Point Scale, 1=Strongly Disagree, 7=Strongly Agree]*
  - *It was easy for me to evaluate this food item.*
  - *It was difficult for me to evaluate this food item. [reverse coded]*
- **Enjoyment of the Experience** *[7 Point Scale, 1=Strongly Disagree, 7=Strongly Agree]*
  - *I enjoyed viewing this food item.*
  - *It was fun to view this food item.*
- **Personal Relevance** *[7 Point Scale, 1=Strongly Disagree, 7=Strongly Agree]*
  - *This is a type of dish I might order when at a restaurant.*
  - *This is similar to other foods I eat.*
- **Psychological Ownership** *[7 Point Scale, 1=Strongly Disagree, 7=Strongly Agree]*
  - *I felt like I had already ordered the Lamb Shawarma.*
  - *I felt like the Lamb Shawarma was already mine.*
- **Willingness to Pay** *[Open Response]*
  - *How much would you be willing to pay (in Pound sterling) to have this Lamb Shawarma right now?*
- **Realism** *[7 Point Scale, 1=Strongly Disagree, 7=Strongly Agree]*
  - *This Lamb Shawarma looks realistic.*
  - *This Lamb Shawarma looks real.*
  - *This Lamb Shawarma looks believable.*
  - *This Lamb Shawarma looks natural.*
- **Mood** *[7 Point Scale, 1=Strongly Disagree, 7=Strongly Agree]*
  - *I am in a good mood right now.*
  - *I am in a bad mood right now. [reverse coded]*

- **AR Familiarity** *[7 Point Scale, 1=Very Unfamiliar, 7=Very Familiar]*
  - *How familiar are you with Augmented Reality technology?*

*In addition, we include measures for PPE (to ensure PPE compliance as part of COVID-19 regulations) and a Tech Check (to account for any technology glitches experienced while using the iPad).

**WEB APPENDIX G**

**Study 3: Full Mediation Analysis on Product Evaluation, Desirability, and Purchase Likelihood**

| **IV** | **Mediator** | **DV: Product Evaluation** | | **DV: Desirability** | | **DV: Purchase Likelihood** | |
| --- | --- | --- | --- | --- | --- | --- | --- |
|  |  | **Effect** | **95% CI** | **Effect** | **95% CI** | **Effect** | **95% CI** |
| Condition | Mental Simulation | **.2809** | **[.0372, .5992]** | **.2559** | **[.0305, .5564]** | **.3560** | **[.0441, .7243]** |
| Condition | Personal Relevance | **.3350** | **[.1210, .6172]** | **.2811** | **[.0850, .5364]** | **.4965** | **[.1851, .8922]** |

**Study 3: Full Sequential Mediation Analysis on Product Evaluation, Desirability, and Purchase Likelihood**

| **IV** | **Mediator** | | **DV: Product Evaluation** | | **DV: Desirability** | | **DV: Purchase Likelihood** | |
| --- | --- | --- | --- | --- | --- | --- | --- | --- |
|  | **1** | **2** | **Effect** | **95% CI** | **Effect** | **95% CI** | **Effect** | **95% CI** |
| Condition | Personal Relevance | Mental Simulation | **.1636** | **[.0418, .3362]** | **.1514** | **[.0388, .3158]** | **.2001** | **[.0549, .4008]** |
| Condition | Mental Simulation | Personal Relevance | **.0734** | **[.0070, .1923]** | **.0614** | **[.0056, .1730]** | **.1093** | **[.0106, .2658]** |

**WEB APPENDIX H**

**Study 4: Food Desirability Pretest**

Fifty participants (44% Female, 54% Male, 2% nonbinary/other; M_Age_ = 39.22, SD = 10.94) recruited from Amazon Mechanical Turk participated in this pretest in exchange for monetary compensation. All participants viewed a static 2D picture of the seven different food items (Waffle Fries, Parmesan Fries, Raw Kale Salad, Chocolate Cake, Fermented Trout, Stewed Eggplant, Fruit and Caramel Crepes) and were asked to rate how desirable they perceived each item to be on three bipolar items (1 = Undesirable and 7 = Desirable). Analysis results indicated that the Parmesan Fries (M = 6.18, SD = 1.26) were considered to be the most desirable food item, with a mean significantly higher than the scale’s mid-point (*t*(49) = 12.27, *p* < .001), while the Fermented Trout (M = 2.52, SD = 1.78) was considered to be the least desirable food item, with a mean significantly lower than the scale’s mid-point (*t*(49) = -5.89, *p* < .001). We accordingly used these as stimuli in our main study.

**Web Appendix I**

**Study 4: Complete List of Measures**

- **Product Evaluation** *[see individual item for scale]*
  - *How desirable did you find the food item to be? [7 Point Scale, 1=Extremely Undesirable, 7=Extremely Desirable]*
  - *How strong is your urge to eat this food item? [7 Point Scale, 1=Extremely Weak, 7=Extremely Strong]*
  - *How strong are your cravings for this food item? [7 Point Scale, 1=Extremely Weak, 7=Extremely Strong]*
  - *After viewing this food item, how likely would you be to order it if it was offered on a menu? [7 Point Scale, 1=Extremely Unlikely, 7=Extremely Likely]*
- **Outcome Mental Simulation** *[7 Point Scale, 1=Strongly Disagree, 7=Strongly Agree]*
  - *I thought about the benefits I would gain from eating this food item*
  - *I thought about why I would eat this food item*
- **Process Mental Simulation** *[7 Point Scale, 1=Strongly Disagree, 7=Strongly Agree]*
  - *I thought about the process of eating this food item*
  - *I thought about how I would eat this food item*
- **Mental Simulation** *[7 Point Scale, 1=Strongly Disagree, 7=Strongly Agree]*
  - *I could imagine myself eating the food item displayed*
  - *I could see myself eating the food item displayed*
  - *I thought about myself eating the food item displayed*
  - *The thought of myself eating this food item felt real*
  - *The thought of myself eating this food item was very clear*
  - *The thought of myself eating this food item was very vivid*
- **Personal Relevance** *[7 Point Scale, 1=Strongly Disagree, 7=Strongly Agree]*
  - *This is a type of dish I might order when at a restaurant*
  - *This is similar to other foods I eat*
- **Realism** *[7 Point Scale, 1=Strongly Disagree, 7=Strongly Agree]*
  - *This food item looks realistic*
  - *This food item looks real*
  - *This food item looks believable*
  - *This food item looks natural*
- **Mood** *[7 Point Scale, 1=Strongly Disagree, 7=Strongly Agree]*
  - *I am in a good mood right now.*
  - *I am in a bad mood right now. [reverse coded]*

- **AR Familiarity** *[7 Point Scale, 1=Very Unfamiliar, 7=Very Familiar]*
  - *How familiar are you with Augmented Reality technology?*
- **Dietary Restriction** *[Yes (please explain), No, Unsure]*
  - *I have a food allergy or dietary restriction which prevents me from consuming this food item.*

**WEB APPENDIX J**

**Study 4: Analysis of AR presentation on Product Evaluation with all participants included**

When including the participant who failed the suspicion probe and the outlier, 2x2 ANOVA revealed a marginally significant main effect of presentation format (*F*(1, 169) = 2.97, M_Control_ = 3.76, M_AR_ = 4.16; *p* = .09, η_p_^2^ = .02) on product evaluation and a significant main effect of food item (*F*(1, 169) = 88.66, M_Undesirable_ = 2.88, M_Desirable_ = 5.00; *p* < .001, η_p_^2^ = .34) on overall product evaluation. The interactive effect was not significant (*F*(3, 169) = .13, *p* = .72, η_p_^2^ = .001).

**WEB APPENDIX K**

**Study 4: Mental Simulation Scale Items Factor Analysis**

The factor analysis (please see the results and component matrix below) demonstrated that while the original mental simulation items and the process-oriented mental simulation items loaded onto the same factor (component 1, below), the two remaining items (both outcome-oriented items) each loaded onto their own separate factors (components 2 and 3).


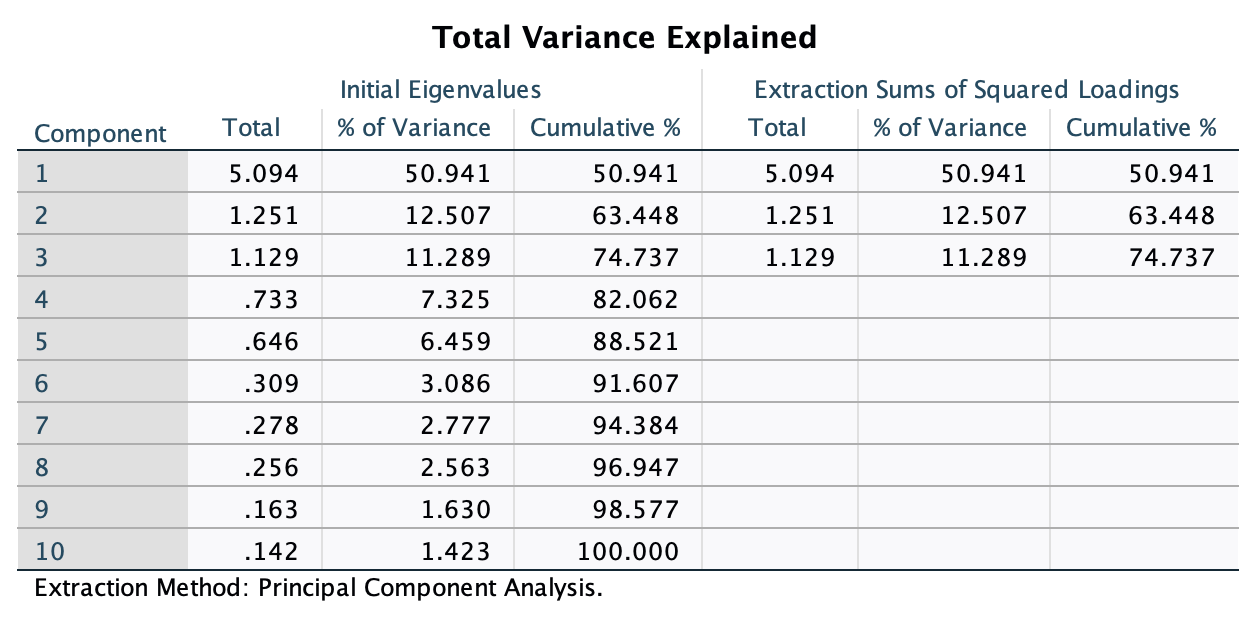


| **COMPONENT MATRIX** | | | | |
| --- | --- | --- | --- | --- |
|  | | **Component** | | |
| **Construct** | **Item** | **1** | **2** | **3** |
| **Outcome Oriented Mental Simulation** | *I thought about the benefits I would gain from eating this food item* | 0.351 | 0.440 | 0.609 |
|  | *I thought about why I would eat this food item* | 0.478 | 0.589 | 0.244 |
| **Process Oriented Mental Simulation** | *I thought about the process of eating this food item* | 0.637 | 0.494 | -0.368 |
|  | *I thought about how I would eat this food item* | 0.662 | 0.417 | -0.428 |
| **Original Mental Simulation Scale** | *I could imagine myself eating the food item displayed* | 0.757 | -0.229 | 0.339 |
|  | *I could see myself eating the food item displayed* | 0.795 | -0.247 | 0.371 |
|  | *I thought about myself eating the food item displayed* | 0.839 | -0.213 | 0.010 |
|  | *The thought of myself eating this food item felt real* | 0.832 | -0.090 | -0.002 |
|  | *The thought of myself eating this food item was very clear* | 0.849 | -0.242 | -0.147 |
|  | *The thought of myself eating this food item was very vivid* | 0.758 | -0.260 | -0.325 |

**WEB APPENDIX L**

**Study 4: Sequential Mediation Analysis Results Using Process-Oriented Mental Simulation Aggregated Scale**

2x2 ANOVA results on the new 8-item process-oriented mental simulation scale revealed a significant main effect of both presentation format (*F*(1, 167) = 3.95, M_Control_ = 4.25, M_AR_ = 4.67; *p* = .05, η_p_^2^ = .02) and food item (*F*(1, 167) = 12.69, M_Undesirable_ = 4.09, M_Desirable_ = 4.83; *p* < .001, η_p_^2^ = .07), while the interactive effect was not significant (*F*(3, 167) = 1.08; *p* = .30, η_p_^2^ = .01). To determine whether the new process-oriented mental simulation scale could replicate the sequential mediation observed by our original scale, we again ran a sequential mediation analysis with 10,000 resamples (PROCESS Model 6, Hayes 2018) with presentation format as the predictor variable, product evaluation as the dependent variable, personal relevance followed by process-oriented mental simulation as the sequential mediators, and food item as a covariate. Results indicated a significant indirect effect of presentation format on product evaluation (indirect effect = .1174, 95% CI: .0361 to .2232).

**WEB APPENDIX M**

**Study 4: Mental Simulation Robustness Checks**

To more rigorously test whether process-oriented (vs. outcome-oriented) mental simulation (MS) represents the underlying mechanism, we created two indices based on the items from the literature (a two-item index for process-oriented MS; *r* = .72; and a two-item index for outcome-oriented MS, *r* = .34). We then ran a series of bootstrapping procedures (each with 10,000 resamples) to test the sequential mediation (model 6; AR 🡪 personal relevance 🡪 mental simulation 🡪 product evaluation) using these indices as the second mediator in the model. In addition, based on the fact that the two outcome-oriented MS items loaded onto separate factors, we ran the same analyses with each of those individual items as the second mediator in the model. The results (along with the previous results reported in our paper) are summarized in the following table:

| **Measure of Mental Simulation (MS)** | **Reliability** | **Indirect effect** | **95% Confidence Interval** |
| --- | --- | --- | --- |
| Original MS Scale  (6 items) | α =.91 | .1272 | [.0379, .2410] |
| Process-Oriented MS Index  (2 items) | r = .72 | .0285 | [.0025, .0722] |
| Outcome-Oriented MS Index  (2 items) | r = .34 | .0102 | [-.0067, .0394] |
| First Outcome-oriented Item  (“I thought about the benefits…”) | NA | .0058 | [-.0099, .0287] |
| Second Outcome-oriented Item  (“I thought about why…”) | NA | .0067 | [-.0066, .0281] |
| Aggregated Process-Oriented MS Scale  (8 items) | α = .90 | .1174 | [0361, .2232] |
